# Oncologic and Reproductive Outcomes After Fertility-Sparing Treatments for Endometrial Hyperplasia with Atypia: A Systematic Review and Meta-Analysis

**DOI:** 10.3390/cancers17243966

**Published:** 2025-12-12

**Authors:** Pál Sebok, Márton Keszthelyi, Balázs Vida, Lotti Lőczi, Barbara Sebők, Petra Merkely, Nándor Ács, Attila Keszthelyi, Szabolcs Várbíró, Balázs Lintner, Richárd Tóth

**Affiliations:** 1 Department of Obstetrics and Gynecology, Semmelweis University, 1082 Budapest, Hungary; sebok.pal.szabolcs@semmelweis.hu (P.S.); vida.balazs.lajos@semmelweis.hu (B.V.); keszthelyi.lotti.lucia@semmelweis.hu (L.L.); merkely.petra@gmail.com (P.M.); acs.nandor@semmelweis.hu (N.Á.); lintner.balazs.zoltan@semmelweis.hu (B.L.); toth.richard@semmelweis.hu (R.T.); 2 Workgroup of Research Management, Doctoral School, Semmelweis University, 1085 Budapest, Hungary; sebok.barbara23@gmail.com (B.S.); varbiroszabolcs@gmail.com (S.V.); 3 Department of Urology, Semmelweis University, 1082 Budapest, Hungary; keszthelyi.attila@semmelweis.hu; 4 Department of Obstetrics and Gynecology, University of Szeged, 6725 Szeged, Hungary

**Keywords:** endometrial hyperplasia, progestin, levonorgestrel-releasing intrauterine device, hysteroscopic resection, GnRHa

## Abstract

**Background**: Atypical endometrial hyperplasia is a precursor of endometrial carcinoma, increasingly diagnosed in reproductive-aged women. Standard hysterectomy may constitute overtreatment. Fertility-sparing approaches, oral or local progestins, hysteroscopic resection, and combined regimens are widely used, though evidence largely derives from early-stage carcinoma. **Methods**: CENTRAL, EMBASE, Scopus, Web of Science, and PubMed were searched from inception to 13 April 2025. Eligible studies included premenopausal women with AEH treated with oral progestins, levonorgestrel intrauterine devices (LNG-IUDs), hysteroscopic resection, or combination regimens (e.g., oral progestins + metformin, LNG-IUD + GnRH analogues). Random-effects meta-analyses with multilevel modeling were applied. Risk of bias was assessed using RoB-2 and ROBINS-I; certainty of evidence was graded with GRADE-PRO. **Results**: Forty-nine studies (2313 women) were included. The pooled complete response (CR) rate was 85% (95% CI 80–89%). LNG-IUDs resulted in 88% CR and oral progestins in 80%. Combination therapies achieved up to 95% CR, though data were limited. Hysteroscopic resection combined with hormonal therapy reported high CR rates (96–97%) but relied on small, heterogeneous cohorts with uncertain reproducibility. The pooled recurrence rate was 19% (95% CI, 13–25%), lower with LNG-IUDs (14%) compared with oral progestins (22%). No response occurred in 14% overall and was lower with LNG-IUDs (13%) than oral progestins (19%). Among women attempting conception, the pooled pregnancy rate was 41%, and the live birth rate was 30%. The mean time to achieve CR was 5.6 months, whereas the mean time to recurrence was approximately 33 months. **Conclusions**: LNG-IUDs provide the most effective and well-supported fertility-sparing treatment for AEH, ensuring high remission with low recurrence. Despite favorable numerical outcomes, the evidence for hysteroscopic resection is limited and inconsistent, not supporting its routine use. Combination regimens may improve response but require confirmation in larger studies.

## Simple Summary

Atypical endometrial hyperplasia is a precancerous condition of the uterine lining that is often grouped together with early endometrial cancer in current guidelines, even though the two differ in how they develop and respond to treatment. Many women diagnosed with this condition wish to preserve fertility, but the effectiveness of available options has remained uncertain. We reviewed 49 studies including 2313 women with atypical endometrial hyperplasia only, allowing us to evaluate outcomes separately from endometrial cancer and provide clearer guidance. Overall, about 85 percent of women achieved complete remission, while around 20 percent experienced recurrence. Treatment with a levonorgestrel-releasing intrauterine device resulted in higher remission and lower recurrence than oral hormone therapy. Among those who tried to conceive, 41 percent became pregnant, and 30 percent had a live birth. Hysteroscopic removal of the endometrium did not show any advantage. These findings may help refine fertility-sparing recommendations.

## 1. Introduction

Atypical endometrial hyperplasia, also termed endometrial intraepithelial neoplasia, is a premalignant precursor of endometrial carcinoma [1]. Approximately 40–50% of cases occur before menopause, and the global incidence is rising in parallel with obesity and metabolic syndrome [1]. AEH progresses to carcinoma in 8% of cases within 4 years and up to 27% in the long term. Furthermore, 40–50% of women harbor concurrent carcinoma at hysterectomy [2,3].

Hysterectomy is the standard treatment of AEH, but fertility-sparing treatments (FSTs), including oral progestins (medroxyprogesterone acetate, megestrol acetate), levonorgestrel-releasing intrauterine devices (LNG-IUDs), hysteroscopic resection (HR), and adjunctive therapies, like metformin or gonadotropin-releasing hormone analogues (GnRHa), are increasingly used [4,5,6].

Reported outcomes vary considerably, particularly in complete response (CR) rate, recurrence rate (RR), pregnancy rate, live birth rate (LBR), partial response (PR) rate, no-response (NR) rate, and mean times to CR and recurrence. The optimal management of AEH in women seeking FST remains unclear due to the lack of AEH-specific guidelines. Current recommendations, including the ESGO/ESHRE/ESGE consensus, focus on early-stage EC and implicitly equate it with AEH [7,8].

Although the ACOG Clinical Consensus No. 5 provides a detailed clinical approach to the management of EIN/AEH, its recommendations remain primarily expert-driven and rely heavily on evidence extrapolated from early-stage EC rather than AEH-specific cohorts [4].

AEH/EIN and early EC are sometimes grouped together, but they differ in invasion status and expected clinical behavior; thus, a shared treatment algorithm is not automatically justified [4]. At the same time, these entities do exhibit substantial overlap in risk factors and genomic features, including PI3K/AKT alterations [9], which contributes to the difficulty of distinguishing them histologically. Diagnostic uncertainty is further compounded by sampling error and intra-tumor heterogeneity, leading to frequent under- or overdiagnosis of occult carcinoma. While guidelines acknowledge these challenges, they provide minimal AEH/EIN-specific directions, reinforcing the need for a focused review.

Considering these limitations, this study presents a systematic review and meta-analysis evaluating FSTs in AEH, examining CR rates, RR, pregnancy and LBR outcomes, PR and NR rates, weighted mean time to CR and recurrence to support individualized care and future evidence-based guidelines.

## 2. Objectives

This systematic review and meta-analysis evaluates the efficacy and safety of fertility-sparing treatments (FSTs) in reproductive-age women with atypical endometrial hyperplasia, comparing oncologic outcomes (response, recurrence, time to CR) and reproductive outcomes (pregnancy rate, live birth rate) across medical and surgical approaches.

## 3. Materials and Methods

### 3.1. Eligibility Criteria, Information Sources, Search Strategy

The following population–intervention–control–outcome (PICO) framework was used:

P—Women of reproductive age, diagnosed with AEH, undergoing FST;

I—Systemic or local progestins, combination therapies (metformin or GnRHa), hysteroscopic resection;

C—Systemic or local progestins, combination therapies (metformin or GnRHa), hysteroscopic resection;

O—CR rate, RR; Pregnancy rate, Live birth rate, PR rate, NR rate, Weighted mean time to CR, Weighted mean time to recurrence.

Studies that included both AEH/EIN and endometrial cancer were eligible only if data for AEH/EIN patients were reported separately. In such cases, we extracted AEH/EIN-specific outcomes exclusively. Studies that did not provide separable AEH/EIN data were excluded.

We systematically searched MEDLINE (via PubMed), Embase, CENTRAL, Web of Science, and Scopus from inception to April 13, 2025, with no restrictions on the initial date of coverage. We applied the following search key using a combination of Medical Subject Headings (MeSH) and free-text terms to capture all relevant studies. The search strategy included the following terms: (“endometrium” OR “endometrial” OR “endometrioid” OR “endometr*”) AND (“hyperplasia” OR “hyperplas*” OR “thick*”) AND (“gestagen” OR “gest*” OR “progesterone” OR “progest*” OR “medroxyprogesterone acetate” OR “medroxyprogesterone” OR “progesterone derivative” OR “megestrol acetate” OR “dienogest derivative” OR “levonorgestrel” OR “hydroxyprogesterone” OR “medrogestone” OR “megestrol” OR “desogestrel derivative” OR “drospirenone” OR “dydrogesterone” OR “intrauterine device” OR “IUD” OR “metformin” OR “GnRHa” OR “gonadotropin-releasing hormone agonist” OR “GnRH analogue” OR “hysteroscopy” OR “hysteros*” OR “hysteroscopic resect*”). The review only included studies published in English. Our analysis comprised both observational studies and randomized controlled trials.

The protocol of the study was registered on PROSPERO (CRD420251031472; 13 April 2025) [10]. The review followed the PRISMA 2020 guideline [11]. (Tables S4 and S5) and the Cochrane Handbook standards (v6.3) [12].

### 3.2. Study Selection

Two independent review authors (P.S., R.T.) selected the articles separately via the EndNote X9 (Clarivate Analytics, Philadelphia, PA, USA) program. Publications were screened based on title and abstract, then the full text was reviewed in Rayyan.ai according to the eligibility criteria. A third, independent review author (P.M.) resolved disagreements during the selection process. The initial search identified 25,619 records, and after removing 3245 duplicates, 22,374 records were screened. Of these, 22,279 were excluded based on title and abstract, and a further 95 after full-text assessment. Including studies identified through citation searching (*n* = 11), a total of 49 articles were selected for inclusion, comprising 36 observational studies and 2 randomized controlled trials, with a combined sample of 2313 patients. Inter-reviewer agreement was high, with Cohen’s Kappa (κ1 = 0.85, κ2 = 0.87) calculated for the first and second steps of the selection, respectively (Supplementary Materials Table S3).

### 3.3. Data Extraction

Two authors (B.V., P.M.) independently extracted study characteristics (author, year, design, treatment, outcomes) into a standardized Excel sheet (Office 365, Microsoft, Redmond, WA, USA). Data on follow-up, treatment duration, comorbidities, and time to CR were collected when available. As progress was rarely reported, partial and no responses were analyzed instead. Outcomes were defined a priori: CR as histological regression to normal endometrium; recurrence as AEH return or progression to carcinoma after CR; pregnancy as at least one confirmed intrauterine pregnancy; live birth as delivery beyond 24 weeks; PR as regression to non-atypical hyperplasia; and NR as persistent or progressive disease. Weighted mean times to CR and recurrence reflected the average intervals to response and relapse.

### 3.4. Assessment of Risk of Bias and Quality of the Evidence

The risk-of-bias assessment in the outcomes was carried out separately by two reviewers, P.S. and B.V., using the Cochrane risk-of-bias tool for randomized trials (RoB 2) [11] and ROBINS-I, a tool for non-randomized studies, visualized using RobVis [13] (Table S2). A third reviewer (M.K.) resolved any disagreements. To assess the quality of the evidence, we followed the recommendations of the “Grading of Recommendations, Assessment, Development and Evaluation (GRADE)” workgroup and assessed the level of evidence certainty using GRADE Pro software (GRADEpro GDT, 2022) [13].

### 3.5. Data Synthesis

We first performed random-effects meta-analyses in a frequentist framework. In our dataset, most studies reported only single-arm outcomes (event/total for one intervention), and only a very limited number of randomized or observational studies provided direct head-to-head comparisons. Therefore, pooled prevalence estimates were used as the primary synthesis method. This approach allowed us to summarize the available evidence across all interventions while avoiding artificial or underpowered comparisons. Odds ratios were extracted or calculated where possible (e.g., in RCTs). Among the few available RCTs [14,15], no statistically significant differences were observed between interventions. Regarding adjusted odds ratios (aORs), we did not identify sufficient information directly related to our predefined outcomes (CR, pregnancy, live birth, partial response, no response) in observational studies. 

For dichotomous outcomes, risk ratios with 95% confidence intervals (CIs) were calculated using the Mantel–Haenszel method, while proportions were pooled separately for each intervention group. For continuous outcomes, either mean differences (MDs) or differences between medians (MedD) were used, depending on data presentation. When only quartile data were reported, means and standard deviations (SDs) were estimated assuming normal or lognormal distributions; where not feasible, medians were pooled instead. Between-study heterogeneity was quantified by Higgins’ I^2^ and τ^2^ estimated using restricted maximum likelihood (REML), with Q-profile CIs. Pooled CIs were adjusted by the Hartung–Knapp method when more conservative estimates were obtained. If ≥8 studies were available, prediction intervals were reported in the forest plots. Sensitivity analyses were performed by sequentially excluding studies at high risk of bias, with small sample sizes (<20 patients), or with extreme results. Subgroup analyses were performed where at least three studies contributed data to a given intervention–outcome category.

To handle correlated outcomes in studies with multiple intervention arms (sometimes assessing the same patients in more than one group), we additionally applied a three-level multivariate random-effects model. This allowed us to incorporate dependency between comparisons without assuming an arbitrary correlation coefficient. Classical two-level models were also repeated to assess robustness and comparability. Small-study effects and publication bias were evaluated by visual inspection of funnel plots and with Peters’ (modified Egger’s) regression or Begg’s test when at least 10 studies were available.

All statistical analyses were performed in R (v4.4.2) using the meta (v7.0.0) package for primary analyses and forest plots, dmetar (v0.1.0) for influence diagnostics, metafor (v4.6.0) for multivariate models, and ggplot2 (v3.5.1) for plotting. Meta-regressions were performed with logit-transformed event rates as the dependent variable and treatment modality as a categorical covariate. Due to insufficient data, we could not meta-analyze weighted mean times to CR or recurrence across all groups; however, for studies reporting medians and ranges/IQR, means and SDs were estimated using established conversion methods. These estimates were applied to derive supplementary pooled values for time to achieve CR (420 patients) and time to recurrence (279 patients). In total, 2313 patients were included in the quantitative synthesis of the meta-analysis [14,16,17,18,19].

## 4. Results

### 4.1. Study Selection

**Figure 1:**
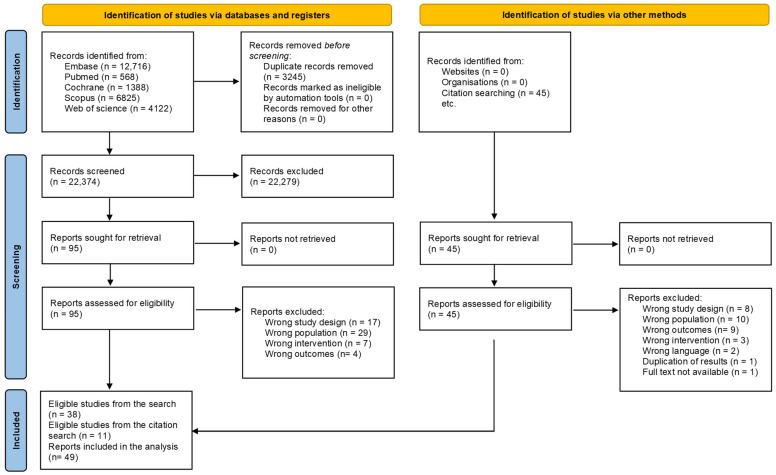
PRISMA 2020 flow diagram of study selection.

The search yielded 25,619 records, of which 49 studies involving 2313 patients met the inclusion criteria (Figure 1).

### 4.2. Study Characteristics

Out of the 49 articles, 37 studies assessed OPs only, 13 assessed IUD only, 3 assessed OPs with HR, 2 assessed IUD with HR, 4 assessed OPs combined with IUD, 3 assessed IUD combined with GnRHa-s, and 7 assessed OPs with metformin. The basic characteristics of the included studies are detailed in Table 1.

### 4.3. Risk of Bias of Included Studies

Most of the included studies were observational with moderate risk of bias, though some were rigorously designed and rated low risk (Supplementary Materials Table S2). Both RCTs [14,15] were prospectively registered in a clinical trials registry prior to participant enrollment. None of the included studies have been retracted.

### 4.4. Synthesis of Results

#### 4.4.1. Complete Response Rates

**Figure 2:**
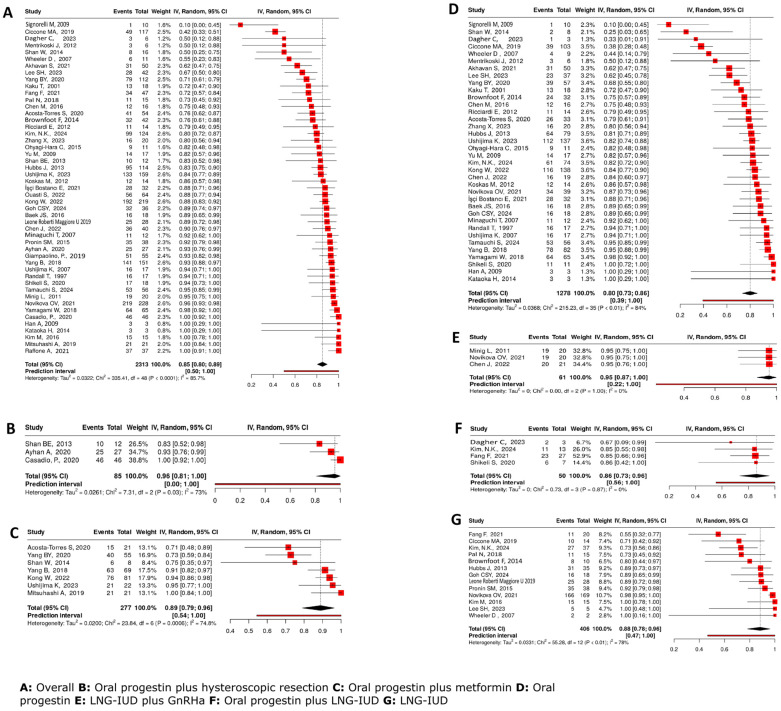
Complete response (CR) rates in women with atypical endometrial hyperplasia treated with fertility-sparing interventions [7,14,15,16,17,18,19,20,21,22,23,24,25,26,27,28,29,30,31,32,33,34,35,36,37,38,39,40,41,42,43,44,45,46,47,48,49,50,51,52,53,54,55,56,57,58,59,60,61]. Red squares: Study-specific complete response rate; square size represents study weight. Horizontal lines: 95% confidence intervals for each study. Black diamond: Pooled effect estimate with its 95% confidence interval. Vertical dotted line: Overall pooled effect estimate (reference line). Heterogeneity statistics: I^2^, τ^2^, and *p*-value describe between-study variability.

Across all included studies [7,14,15,16,17,18,19,20,21,22,23,24,25,26,27,28,29,30,31,32,33,34,35,36,37,38,39,40,41,42,43,44,45,46,47,48,49,50,51,52,53,54,55,56,57,58,59,60,61], the overall pooled CR rate to FSTs for AEH was 85% (95% CI: 80–89%) with high heterogeneity (I^2^ = 85.7%) (Figure 2).

#### 4.4.2. Oral Progestins Alone

37 studies [7,14,15,16,17,19,20,21,24,25,26,27,28,29,30,31,33,35,38,39,41,42,43,44,45,46,47,48,49,50,53,54,55,57,58,60] covering 1342 patients assessed OPs; the pooled CR rate was 80% (95% CI: 74–86%), with considerable heterogeneity (I^2^ = 83.5%).

#### 4.4.3. LNG-IUD Alone

The use of LNG-IUD showed a high CR rate of 88% (95% CI: 78–96%) in 13 studies [15,20,22,25,29,30,39,40,43,46,51,55,61] covering 406 patients with high variability across studies (I^2^ = 78%).

#### 4.4.4. Combination Therapies with HR

3 studies [18,23,36] covering 85 patients assessed treatment with OPs plus hysteroscopic endometrial resection, achieving a CR rate of 96% (95% CI: 81–100%). 2 studies [36,52] covering 92 patients assessed LNG-IUD after receiving HR, achieving a CR of 97% (95% CI: 86–100%); however, the small sample size limited statistical power and generalizability.

#### 4.4.5. Combined Methods of Conservative Therapies

Seven studies [7,14,19,34,41,45,59] covering 277 patients assessed OPs plus metformin (Figure 2C), achieving a CR rate of 89% (95% CI: 79–96%). The combination of LNG-IUD and a GnRH analogue demonstrated a high CR rate of 95% (95% CI: 87–100%), with no observed heterogeneity (I^2^ = 0%) based on three studies [27,30,32] covering 61 patients. OP combined with LNG-IUD yielded a CR rate of 86% (95% CI: 73–96%) from four studies [25,26,29,47] including 100 patients with no heterogeneity detected (I^2^ = 0%) (Figure 2).

#### 4.4.6. Recurrence Rate

**Figure 3:**
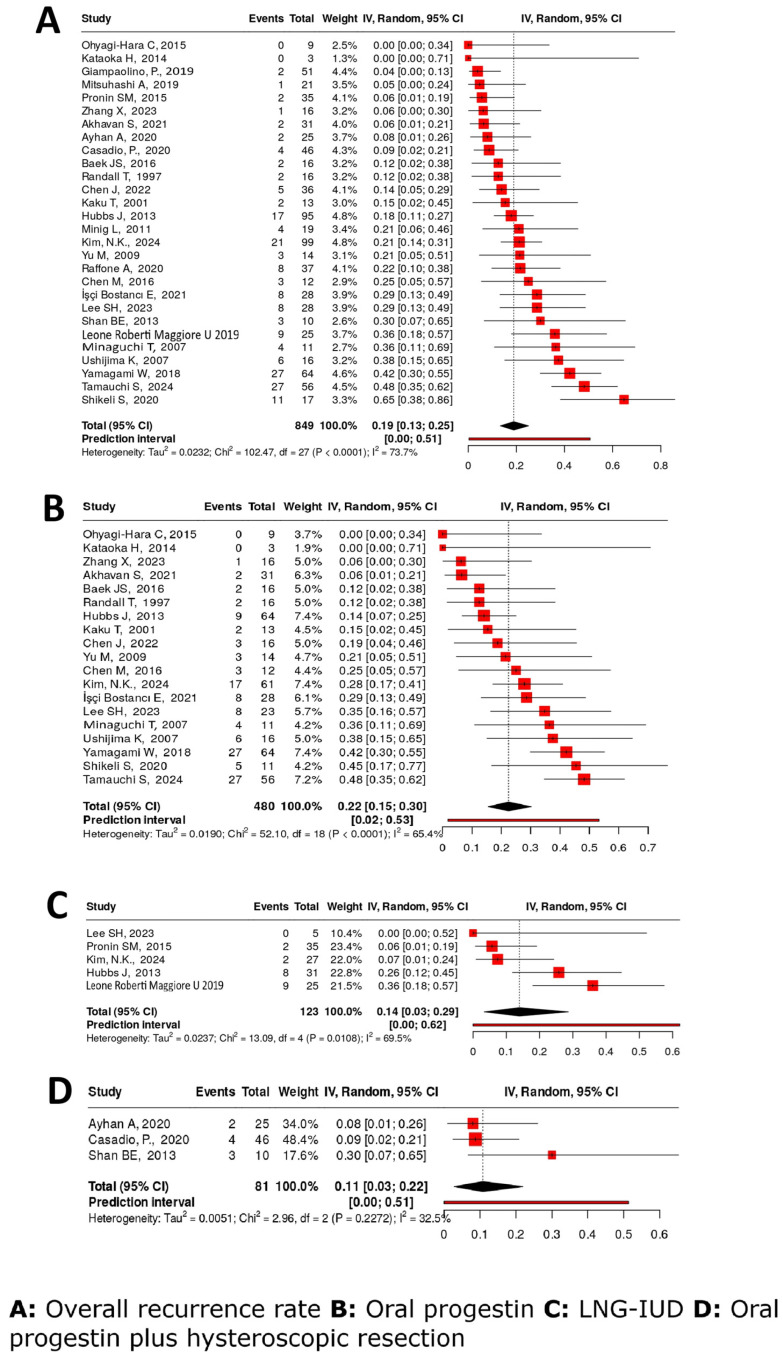
Recurrence rates after fertility-sparing treatment [16,17,18,21,22,23,24,25,26,27,28,31,32,33,34,36,37,38,39,40,42,43,44,52,53,56,57,58].

Twenty-eight studies [16,17,18,21,22,23,24,25,26,27,28,31,32,33,34,36,37,38,39,40,42,43,44,52,53,56,57,58] assessed RR, yielding a pooled RR of 19% (95% CI: 13–25%), with substantial heterogeneity (I^2^ = 73.7%). OP monotherapy, evaluated in 19 studies [16,17,21,24,25,26,27,28,31,33,38,39,42,43,44,53,56,57,58], was associated with a higher RR of 22% (95% CI: 15–30%) (I^2^ = 65.4%). OPs combined with HR [18,23,36] demonstrated a significantly lower RR of 11% (95% CI: 3–22%), with minimal heterogeneity (I^2^ = 32.5%). The recurrence rate of LNG-IUD [22,39,40,43] was 14% (95% CI: 3–29%), with substantial heterogeneity (I^2^ = 69.5%) (Figure 3).

#### 4.4.7. Pregnancy Rate

**Figure 4:**
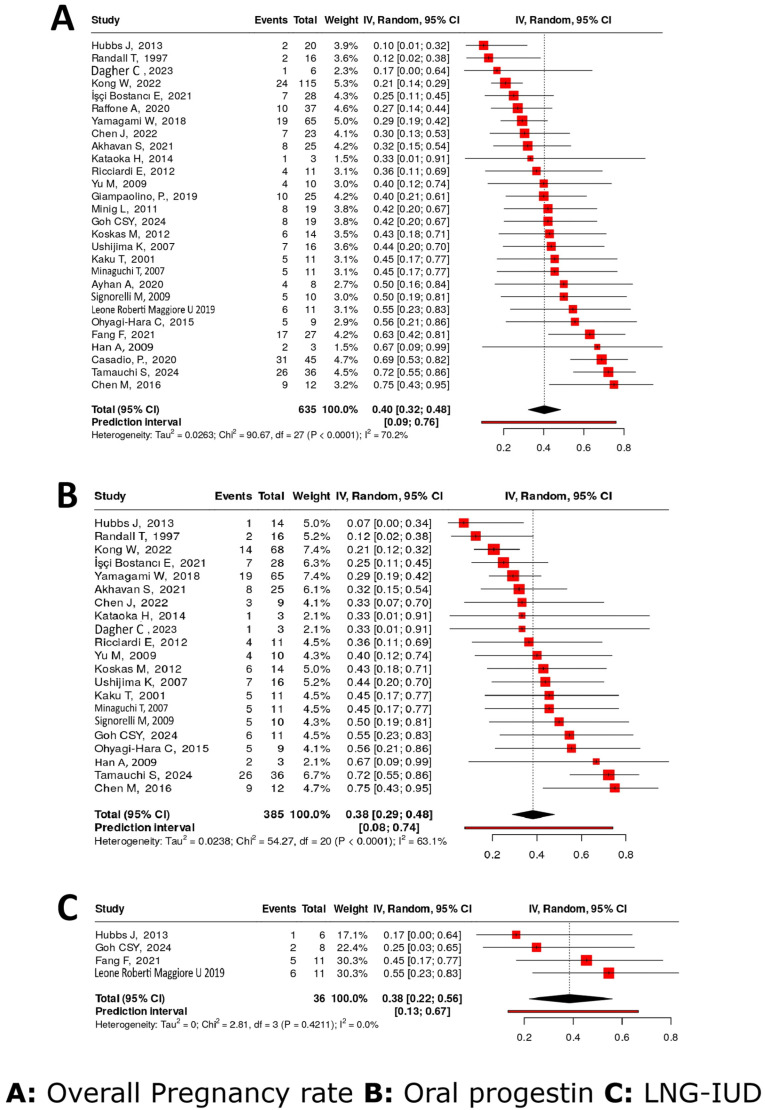
Pregnancy rate [15,16,17,18,21,24,27,28,29,31,32,33,36,37,38,40,43,44,45,47,48,49,52,53,54,56,58,60].

Twenty-eight studies [15,16,17,18,21,24,27,28,29,31,32,33,36,37,38,40,43,44,45,47,48,49,52,53,54,56,58,60] were included, reporting on pregnancy rates of 631 women. The overall pooled pregnancy rate following FST for AEH was 41% (95% CI: 33–49%), with substantial heterogeneity (I^2^ = 70.7%). In 21 studies, where patients were treated with OP monotherapy [15,16,17,21,24,27,28,31,33,38,43,44,45,47,48,49,53,54,56,58,60], the pregnancy rate was 39% (95% CI: 29–49%), with moderate heterogeneity (I^2^ = 64.2%). Pooled data from four studies evaluating LNG-IUD monotherapy [15,29,40,43] showed a pregnancy rate of 38% (95% CI: 22–56%), with no observed heterogeneity (I^2^ = 0.0%) (Figure 4).

#### 4.4.8. Live Birth Rates

Twenty-three studies [15,16,17,18,21,24,27,29,31,32,33,36,37,38,40,43,44,45,47,48,49,53,58] covering 485 patients assessed various FSTs for AEH and their associated LBRs. The pooled LBR across all treatment modalities was 30% (95% CI: 23–38%), with moderate heterogeneity observed (I^2^ = 61.5%).

**Figure 5:**
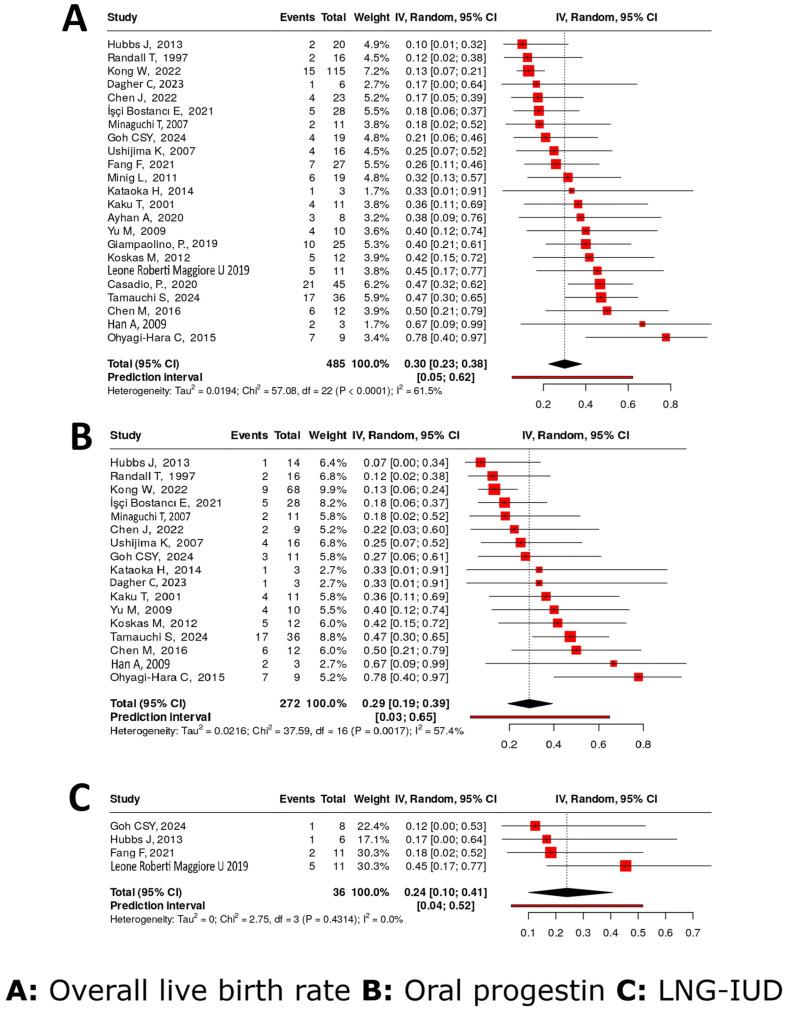
Live birth rate [15,16,17,18,21,24,27,29,31,32,33,36,37,38,40,43,44,45,47,48,49,53,58].

Seventeen studies [15,16,17,21,24,27,31,33,38,43,44,45,47,48,49,53,58] including 272 patients evaluated OPs alone, yielding a LBR of 29% (95% CI: 19–39%). Moderate heterogeneity was present (I^2^ = 57.4%). Four studies [15,29,40,43] involving 36 patients examined the use of LNG-IUD alone, showing an LBR of 24% (95% CI: 10–41%), with no observed heterogeneity (I^2^ = 0%) (Figure 5).

#### 4.4.9. Partial Response Rate

Fourteen studies [16,21,24,28,29,33,35,37,40,50,51,55,57,59] including 378 patients evaluated PR, yielding a pooled rate of 8% (95% CI: 5–12%), with no observed heterogeneity (I^2^ = 0%). Ten studies [16,21,24,28,33,35,50,55,57,59] covering 225 patients assessed OPs only, with a PR rate of 10% (95% CI: 6–15%) and no heterogeneity (I^2^ = 0%). Three studies [29,40,51] comprising 63 patients evaluated LNG-IUD monotherapy, demonstrating a PR response rate of 9% (95% CI: 3–18%) with no heterogeneity (I^2^ = 0%) (Figure S1).

#### 4.4.10. No-Response Rate

The pooled no-response rate of twenty studies [15,17,18,21,22,23,28,29,33,35,37,38,40,50,51,53,55,57,59,60] covering 571 patients was 14% (95% CI: 9–21%) with high heterogeneity observed (I^2^ = 73.4%).

Thirteen studies [15,17,21,28,33,35,38,50,53,55,57,59,60] including 326 patients examined OPs alone, reporting an NR rate of 19% (95% CI: 10–31%), with substantial heterogeneity (I^2^ = 79.2%).

Five studies [15,22,29,40,51] involving 116 patients assessed the use of LNG-IUD alone, resulting in an NR rate of 13% (95% CI: 4–25%), with moderate heterogeneity (I^2^ = 62.7%) (Figure S1).

#### 4.4.11. Weighted Mean Time to CR and Weighted Mean Time to Recurrence

Across studies with available or estimable means ± SD, the weighted mean time to achieve CR was 5.6 ± 6.7 months (95% CI: 5.0–6.3; 420 patients). The weighted mean time to recurrence was 32.7 ± 11.4 months (95% CI: 31.3–34.0; 279 patients).

### 4.5. Assessing the Certainty of Evidence

Overall, most studies were methodologically sound; however, the primary limitations were small sample sizes and the predominance of retrospective study designs. Quality ratings were higher for frequently reported, well-established outcomes and lower for outcomes supported by limited, highly similar, or heterogeneous evidence (Supplementary Materials Table S1).

### 4.6. Influencing Factors

Although BMI and age were consistently reported across studies, both variables exhibited minimal between-study variability, making them unlikely contributors to statistical heterogeneity. Progestin dose regimens varied widely, but reporting was inconsistent and often lacked sufficient detail for quantitative analysis. Even when doses were reported, they frequently appeared as broad, diverse ranges, precluding meaningful dose–response exploration. Molecular markers were reported in only a few studies, and other key clinical factors, such as insulin resistance, PCOS, or treatment adherence, were also inconsistently documented. As a result, their potential contribution to heterogeneity could not be assessed.

Because total follow-up duration could theoretically affect recurrence detection, we performed an exploratory stratified analysis comparing studies with shorter (<24 months) versus longer (≥24 months) follow-up. Recurrence rates and heterogeneity were highly similar across both strata, indicating that differences in follow-up length did not materially contribute to between-study variability, although shorter observation periods may still underestimate late recurrences (see Supplementary Materials Figure S3). Moreover, because our outcomes were proportion-based rather than time-to-event measures, follow-up duration could not be appropriately incorporated into meta-regression. Importantly, sensitivity analyses excluding small studies, statistical outliers, and high-risk studies yielded stable pooled estimates, further confirming that these factors did not materially influence our results (Table 1).

### 4.7. Assessment of Reporting Bias

No substantial asymmetry was observed for CR and recurrence, suggesting low reporting bias, while for pregnancy, LBR, PR, and NR, the small number of studies limited interpretation. Moreover, many included studies originated from single centers or highly specialized fertility units, which may limit the broader applicability and generalizability of the findings (Supplementary Materials Figure S2).

### 4.8. Sensitivity Analysis

According to the RoB (robvis) assessment, no study was rated as “high risk.” Therefore, no sensitivity analysis was required for this criterion.

When studies with fewer than 20 patients were excluded, results were largely consistent with the original pooled estimates. For the CR rate, the pooled odds ratios remained stable (overall OR 0.85 vs. 0.87), although heterogeneity slightly increased. Subgroup analyses generally showed similar patterns, except for subgroups where exclusion of small studies left only a single study (e.g., OP + IUD), in which case pooled estimates were not feasible. For outcomes such as pregnancy and LBR, some subgroups could not be analyzed further because all available studies included fewer than 20 patients.

Exclusion of statistical outliers did not materially alter the direction of the results but led to reduced heterogeneity across most outcomes. For example, in the overall complete response analysis, I^2^ decreased from 85.7% to 72.9% after removal of outliers, while the pooled effect size (OR 0.85 vs. 0.86) remained consistent. Similarly, for pregnancy rate and live birth rate, heterogeneity was substantially reduced (from 70–80% to below 40% or even 0%), while pooled ORs were essentially unchanged.

Overall, the sensitivity analyses confirmed the robustness of the main findings. Exclusion of small studies or outliers did not significantly change the effect estimates, although heterogeneity was somewhat reduced in certain outcomes. In subgroups where only very small studies were available, sensitivity analyses could not be conducted, and results should be interpreted with caution.

Notably, only one included study was published before 2000, whereas the vast majority appeared after 2010, when AEH/EIN diagnostic criteria had largely converged, reducing the likelihood of meaningful variability in diagnostic definitions. Furthermore, follow-up protocols were highly consistent across studies, with histological reassessment typically performed at approximately 3-month intervals. Given this relative uniformity, neither diagnostic nor follow-up heterogeneity is likely to have materially influenced the pooled estimates, a conclusion supported by the stability of the results across all sensitivity analyses.

## 5. Discussion

This systematic review and meta-analysis presents a focused and disease-specific analysis of fertility-sparing treatments for atypical endometrial hyperplasia (AEH). The European ESGO/ESGE/ESHRE consensus guidelines emphasize fertility preservation for early EC but offer only general recommendations for AEH, despite the biological, histological, and clinical differences between them. Our study addresses this gap by exclusively evaluating AEH, providing clearer, more applicable data for clinical decision-making in women with this premalignant condition.

A major strength of our study lies in its exclusive focus on patients with atypical endometrial hyperplasia (AEH). This disease-specific approach enhances the clinical relevance and accuracy of our estimates. In contrast, several meta-analyses, including those by Wei et al. [16], Li et al. [62] and Zhao et al. [63], analyzed mixed cohorts of AEH and EC, thereby diluting AEH-specific outcomes with data from more advanced or biologically distinct cases. For example, Wei et al. [64] reported a pooled complete response (CR) rate of 71% for oral progestins in a mixed population, substantially lower than our AEH-only CR of 84%, likely due to the inclusion of EC cases with more aggressive behavior. Li et al. similarly found an overall CR of 75.8% and a live birth rate of 26.8%, again without stratifying by diagnosis.

By limiting our analysis to AEH, we provide cleaner, more targeted estimates that better reflect the natural history and treatment responsiveness of this premalignant but non-invasive condition.

Our study found no overall statistically significant differences in complete response rate, recurrence rate, pregnancy rates, live birth rate, no-response rate, or partial response rate across treatment modalities in AEH when considering overlapping confidence intervals. CR rates were 80% for oral progestins, 88% for LNG-IUD, and up to 95% for combination therapies, including metformin or GnRH analogues. Meta-regression analysis indicated that the combination of oral progestins with hysteroscopic resection was associated with a significantly higher CR rate compared to oral progestins alone (*p* = 0.041); however, this result is based on a small number of studies (n = 3) and should be interpreted with caution due to the limited sample size (n = 85). Similarly, pregnancy rates showed no significant overall differences; however, oral progestins combined with hysteroscopic resection were associated with a modestly higher pregnancy rate. This finding was based on a small number of reports and relatively low sample sizes and should be interpreted with caution. No significant differences were observed for no-response rates, although combinations such as IUD + GnRH analogues and IUD + hysteroscopic resection showed trends toward lower rates than other regimens. For PR, the combination of oral progestins with metformin yielded a significantly lower partial response rate compared to IUD (*p* <0.001); however, this estimate was based on a single study with a small sample size (n = 8) and zero events and should therefore be interpreted with caution. These results suggest that while broad comparisons may not reach statistical significance, certain combinations could offer clinical advantages or disadvantages. This is consistent with earlier literature: Wei et al. [64] reported CR rates of 71% for oral progestins, 76% for LNG-IUD, and 87% for the oral + IUD combination in a mixed AEH/EC population—suggesting a potential benefit from combined regimens, though the presence of EC cases complicates interpretation. Gallos et al. [65] reported a pooled CR of 85.6% in AEH patients across treatments, supporting the general effectiveness of progestin-based conservative management regardless of delivery route. Overall, while broad comparisons did not show significance, certain combinations may offer clinical advantages. Emerging evidence suggests that molecular classification may help identify subgroups with differential responses to fertility-sparing therapy. Ferrari et al. recently demonstrated that POLE-mutated and MMRd (Mismatch Repair Deficiency) tumors exhibit distinct oncologic behavior during conservative management, although AEH-specific validation remains limited [66].

Several studies have explored the addition of metformin to progestin therapy, motivated by its anti-proliferative and insulin-sensitizing effects, particularly in patients with metabolic comorbidities such as PCOS or obesity. In our AEH-only analysis, oral progestin combined with metformin achieved a CR rate of 89% (95% CI: 79–96%), outperforming oral progestin alone (80%) and comparable to other combination therapies; however, no significant difference was measured. In an AEH-only subset, Shao et al. [67] showed a CR of 79.4% with oral progestin monotherapy and 87.1% with progestin plus metformin, closely mirroring our own findings. In contrast, Factor et al. [68] concluded that the addition of metformin provided no significant benefit in CR or LBR for AEH or EC patients, although their findings were more relevant to non-atypical hyperplasia and lacked stratification by disease subtype. Accordingly, Adamyan et al. [69] found no statistically significant difference between progestin alone and progestin plus metformin in pooled analyses of AEH and EC. Metformin showed superiority to the standard regimen in achieving a better CR rate in patients with AEH. In terms of reproductive outcomes, combined therapy benefits pregnancy rates but not recurrence or live birth rates.

While previous studies have suggested that GnRH analogues may enhance the efficacy of fertility-sparing treatments, our analysis indicates that their added benefit is modest and context-dependent. De Rocco et al. [70] reported a low pregnancy rate of 15.4% and no live births when GnRH analogues were combined with LNG-IUD or letrozole, highlighting concerns about effectiveness. In contrast, Fan et al. [71] observed a high CR rate of 72.9% (95% CI: 60.4–82.5%) in patients treated with LNG-IUS combined with GnRH analogue or progestin. Consistent with these findings, our analysis found a 95% CR rate for the combination of LNG-IUD and GnRH analogue. However, the clinical use of GnRH analogues is tempered by significant side effects—particularly hypoestrogenism and bone loss—which limit their utility in prolonged treatment courses typical for AEH. Therefore, while CR rates appear promising in selected combinations, the role of GnRH analogues remains best reserved for tailored or short-term protocols.

One of the most critical endpoints for patients undergoing fertility-sparing therapy for AEH is the likelihood of achieving a live birth. While our pooled pregnancy rate of 41% indicates that conception is achievable in a substantial proportion of patients undergoing fertility-sparing treatment for AEH, the rate remains modest considering the reproductive intent of these therapies. Notably, both oral progestin and LNG-IUD monotherapies yielded comparable pregnancy rates (39% and 38%), despite differences in route of administration and pharmacokinetics. The lack of significant heterogeneity among LNG-IUD studies (I^2^ = 0%) suggests greater consistency in reproductive outcomes, possibly due to improved compliance and more uniform endometrial exposure compared to oral agents. In our meta-analysis, we found a pooled live birth rate (LBR) of 30% (95% CI: 23–38%) across all interventions. When stratified by modality, oral progestin monotherapy yielded a 29% LBR, while LNG-IUD monotherapy resulted in a 24% LBR. Combination therapies, such as oral progestins with metformin or LNG-IUD with GnRH analogues, showed a modest increase, with live birth rates ranging from 33% to 42%; however, as fewer than three studies reported results for each regimen, we did not analyze these statistically as separate subgroups. Confidence intervals overlapped, and statistical comparisons did not reveal a definitive advantage.

Our findings are in line with several earlier meta-analyses, although many of those studies combined AEH and EC populations. For instance, Wei et al. [64] reported a live birth rate of 20% with oral progestins and 14% with LNG-IUDs in a mixed cohort, while the IUD + oral combination achieved a slightly higher 35%. Similarly, Li et al. [62] found a pooled pregnancy rate of 26.8%, with a live birth rate of 73.8% among those who conceived, but without stratifying by treatment type or AEH-only cases.

One of the most detailed analyses was conducted by De Rocco et al. [70], who reported live birth rates (LBRs) of 43/53 (80.8%) with LNG-IUD alone, 136/167 (81.0%) with oral progestins, 37/53 (69.9%) with oral progestin plus metformin, and 32/45 (69.9%) with LNG-IUD plus oral agents. Although these rates appear higher than ours, it is important to note that De Rocco included both AEH and early-stage EC cases, and their LBRs were calculated from treatment responders, not stratified by disease subtype, making direct comparisons limited.

In contrast, Zhao et al. [63] reported an AEH-specific LBR of 22.2% overall and 23.9% among complete responders, closely matching our pooled LBR of 30%. These results support the notion that, despite high rates of histologic remission, fewer than one-third of women ultimately achieve live birth—likely due to factors such as age, comorbidities, infertility background, and limited ART access.

Not all studies supported a benefit of combination or intensified therapy. For example, Adamyan et al. [69] found no significant difference in fertility outcomes between standard hormonal therapy and regimens augmented with metformin across mixed AEH and EC cohorts. Similarly, Factor et al. [68] concluded that metformin provided no added reproductive benefit in atypical hyperplasia or EC. These findings align with our observation that while combination therapies may trend toward higher LBR, the differences are not statistically robust.

Overall, the variability in live birth rates across studies—ranging from 14% to over 50% depending on population and definitions—underscores the importance of evaluating AEH separately from EC. Our AEH-only analysis provides a more disease-specific estimate and suggests that progestin-based therapies, regardless of delivery route, result in similar reproductive potential. Importantly, these results emphasize that oncologic remission does not guarantee fertility success, and multidisciplinary planning—including fertility counseling and potential referral for ART—should be integral to care for women pursuing fertility preservation in AEH.

Regarding treatment failure, our pooled non-response rate was 14%, with oral progestins showing a higher failure rate of 19% compared to 13% for LNG-IUD monotherapy. This modest difference supports prior findings that intrauterine delivery systems may offer more consistent endometrial exposure and improved compliance. In line with our findings, Wei et al. [64] reported a non-response rate of approximately 29% for oral progestins and 24% for LNG-IUDs in a mixed AEH/EC cohort. Fan et al. [71] observed a lower recurrence rate of 11% with IUD-based regimens compared to 30.7% with oral progestins alone in early EC, indirectly suggesting lower non-response with intrauterine approaches. Zhao et al. [63] found a 10.3% non-response rate in AEH patients treated with hysteroscopic resection plus progestin, slightly lower than our pooled result, though their population and intervention differ in scope.

Partial response (PR) rates were consistently low across all studies. In our analysis, PR occurred in 11% of patients treated with oral progestins and 9% of those receiving LNG-IUDs. Notably, very few prior meta-analyses reported partial response as a distinct outcome. This omission limits direct comparison but highlights the added granularity of our analysis.

In this meta-analysis, the estimation of mean and SD values was derived from medians and ranges/IQR, allowing pooled time-to-event metrics for a subset of the population. The weighted mean time to CR was 5.63 months and to recurrence 32.65 months, providing insight into treatment duration. However, as these estimates represent only a portion of our total cohort (420 and 279 out of 2313 patients, respectively), they should be interpreted with caution. These limitations highlight the need for standardized reporting of time-to-event data in future studies.

Hysteroscopic endometrial resection has been extensively evaluated in the management of early-stage endometrial cancer (EC), where it is often combined with progestin therapy as part of fertility-preserving treatment protocols. Several high-quality meta-analyses, particularly those by Fan et al. [71], Zhao et al. [63], Ye et al. [72] and Suzuki et al. [73], have reported encouraging oncologic and reproductive outcomes in these EC-focused populations. They demonstrated CR rates as high as 95.3% in EC using oral progestins with hysteroscopic resection, although AEH-specific subgroup data were not provided. For instance, Fan et al. found a complete response (CR) rate of 95.3%, recurrence rate (RR) of 14.1%, and pregnancy rate of 47.8% in patients with stage IA, grade 1 EC treated with hysteroscopic resection plus progestins [71]. Similarly, Zhao et al. reported a CR of 97% for AEH (compared to 88.6% for EC), with recurrence rates of 10.3% in AEH and 18.3% in EC, and live birth rates of 22.2% in AEH and 26% in EC [63]. In a separate study, Ye et al. found 100% CR in AEH and 90% in EC, with AEH-specific live birth and pregnancy rates of 44% and 47%, respectively [72].

Despite these strong results in EC, the translatability of such outcomes to AEH remains highly questionable. The pathophysiological distinction is essential: EC typically arises as a focal, often polypoid lesion amenable to targeted resection, while AEH presents as a diffuse and multifocal glandular abnormality, making complete hysteroscopic excision technically impractical and histologically unreliable. This distinction has been emphasized in standard gynecologic pathology references, including Ellenson et al. where AEH is described as a “field change” rather than a localized neoplasm [74].

Our findings do not provide sufficient evidence to support the routine use of hysteroscopic resection in AEH, given the small, heterogeneous cohorts and the diffuse nature of the disease. With progestin-based therapies, particularly LNG-IUD, we achieved a pooled CR of 88% and a live birth rate of 24%, comparable to outcomes from hysteroscopy-assisted treatments in EC, despite our AEH-only focus. Partial response rates remained low (~9–11%), confirming the effectiveness of medical therapy. Moreover, recent reviews by Bilir et al. and Ye et al. highlight the lack of AEH-specific data and limited accessibility of high-quality hysteroscopy [72,75]. In addition, many studies evaluating hysteroscopic resection or combination regimens included very small AEH cohorts, which further limit statistical power and reduce confidence in the reliability of these estimates.

Taken together, these data suggest that hysteroscopic resection offers no clinically meaningful advantage in the treatment of AEH. Given its diffuse histologic nature, the lack of consistent AEH-specific outcome data, and the procedural expertise required, hysteroscopy should not be routinely recommended in this population. These observations reinforce the need to distinguish AEH from EC in both clinical research and treatment guidelines. Instead, LNG-IUD-based regimens, either alone or in combination with oral agents, represent effective, accessible, and well-tolerated alternatives—particularly in settings where surgical expertise or infrastructure is limited.

### Strengths and Limitations

This meta-analysis has several notable strengths. It is one of the few systematic reviews focusing exclusively on AEH, providing disease-specific oncologic and reproductive outcomes. The clear separation from EC enhances the clinical relevance. Additional strengths include a pre-registered protocol, PRISMA adherence, advanced multilevel modeling, and a large, pooled sample enabling detailed subgroup analyses.

Several limitations must be acknowledged in this meta-analysis. Most included studies were observational, and although efforts were made to assess and adjust for bias, inherent variability in study design, sample sizes, patient characteristics, and follow-up durations likely contributed to statistical heterogeneity. Although most studies applied standardized AEH/EIN diagnostic frameworks, slight variation in the formal criteria used (WHO 1994, WHO 2014, EIN) [76,77,78] may have introduced minor conceptual heterogeneity. These diagnostic criteria share substantial morphological overlap, and none of the included studies applied definitions that differed in any clinically meaningful way. Several studies tended to allocate specific fertility-sparing treatments to younger or healthier women, or to those with better reproductive prognoses. This treatment-allocation bias may have overstated the apparent effectiveness of some regimens.

Many studies combined atypical endometrial hyperplasia with endometrial carcinoma without clear distinction, requiring the exclusion of inadequately stratified data to avoid misclassification bias and reducing the analyzable evidence base. Furthermore, outcomes were rarely stratified by important clinical variables, including BMI, metabolic status, insulin resistance, PCOS, or infertility history. The absence of these data limits subgroup interpretation and may have affected both oncologic and reproductive outcomes, helping to explain the heterogeneity observed.

The absence of standardized hormonal protocols likely introduced confounding and reduced comparability between cohorts, and although follow-up intervals varied, histologic reassessment was typically performed every three months, making a meaningful impact on results unlikely. While total follow-up duration varied across studies and could theoretically affect the detection of late recurrences, our exploratory analyses indicated that differences in follow-up length did not materially alter recurrence estimates or contribute meaningfully to between-study variability. Nonetheless, shorter observation periods may still underestimate delayed recurrences, underscoring the importance of long-term surveillance in future AEH research. Furthermore, most included studies were conducted in single-center or specialized fertility units, which reflects the expertise required for these treatments but may limit the generalizability of our findings to broader clinical settings.

Reproductive outcomes were frequently underreported or incompletely characterized. Many studies did not distinguish between spontaneous and ART-assisted conceptions, and only a subset of women actively attempted pregnancy, making pregnancy and live birth rates potentially unrepresentative of the entire treated population. Publication bias is also possible, as studies reporting more favorable oncologic or reproductive outcomes may be more likely to be published, potentially overestimating treatment efficacy.

Finally, although several outcomes exhibited substantial heterogeneity, consistent reporting of variables such as progestin dose, treatment duration, BMI, metabolic markers, or molecular profiles was insufficient to support meta-regression or informative stratified analyses. These variables were either too sparsely or too inconsistently reported or showed minimal between-study variability, preventing meaningful modeling of their contribution to heterogeneity. Time-to-event outcomes were available only for a minority of patients, further limiting precision. Together, these limitations highlight the need for prospective, AEH-specific studies with standardized diagnostic criteria, uniform reporting of treatment protocols and reproductive endpoints, comprehensive collection of relevant clinical covariates, and adequate long-term follow-up.

## 6. Conclusions

This AEH-specific meta-analysis provides the most robust evidence to date on the oncologic and reproductive outcomes of fertility-sparing therapies in reproductive-aged women. Our results support LNG-IUD-based therapy as the most effective and practical fertility-sparing strategy, achieving high CR rates with a favorable safety profile. Although oral progestin monotherapy remains widely used, our findings suggest it may be suboptimal, showing lower CR and higher non-response rates, suggesting limited efficacy as a monotherapy. Combination therapies, such as oral progestins with metformin or LNG-IUD with GnRH analogues, achieved higher CR rates, although live birth rates remained marginal and statistically nonsignificant. Importantly, hysteroscopic resection, while effective in early endometrial cancer, does not demonstrate additional benefit over medical therapy for AEH due to its diffuse, multifocal histology. The evidence does not support its routine use in AEH management, and procedural limitations further reduce its practicality, especially in settings lacking surgical expertise. Clinicians should consider LNG-IUD as first-line therapy, either alone or in combination, tailored to individual patient characteristics, and ensure timely referral for fertility counseling or assisted reproductive technologies when appropriate. Importantly, current clinical guidelines do not clearly delineate AEH from early-stage endometrial cancer in their management recommendations. Given the distinct natural history and response patterns of AEH, it is imperative that future iterations of international guidelines, such as those from ESGO, ESHRE, and ESGE, develop specific diagnostic and therapeutic algorithms tailored to AEH. Future research should aim to generate robust, AEH-specific data through prospective, controlled studies with standardized hormonal protocols and clearly defined fertility endpoints. Such studies are essential to refine clinical decision-making and to support the development of dedicated treatment guidelines for atypical endometrial hyperplasia that are distinct from those for early endometrial cancer.

## Figures and Tables

**Table 1:** Basic characteristics of included studies.

Study	No. of Patients	Age ^1^	BMI ^2^	Therapy ^3^	Regimen ^4^	CR ^5^	Recurrence Rate ^6^	Pregnancy Rate ^7^	Live Birth Rate ^8^	Partial Response ^9^	No Response ^10^
Ushijima K 2007 [16]	17	31.7 M	22.8 M	Oral	MPA 600 mg	16/17	6/16	7/16	4/16	1/17	N/A
Bostancı İ E 2021 [17]	32	43.78 M	32.85 M	Oral	MA 80–480 mg	28/32	8/28	7/28	5/28	N/A	4/32
Ayhan A 2020 [18]	27	34 (20–43) MD	28.8 (22–41) m	Oral + HR	MA 160 mg	25/27	2/25	4/8	3/8	N/A	2/27
Acosta-Torres S 2020 [19]	54	37.7 MD	35 m	Oral	MA 80–160 mg, MPA 10–40 mg	26/33	N/A	N/A	N/A	N/A	N/A
		37.7 MD	35 m	Oral + Met	MA 80–160 mg, MPA 10–40 mg, Met 500–1000 mg	15/21	N/A	N/A	N/A	N/A	N/A
Yang BY 2020 [14]	112	N/A	N/A	Oral	MA 160 mg	39/57	N/A	N/A	N/A	N/A	N/A
		N/A	N/A	Oral + Met	MA 160 mg, Met 1500 mg	40/55	N/A	N/A	N/A	N/A	N/A
Ciccone MA 2019 [20]	117	35 M	40.2 M	Oral	MA, MPA	39/103	N/A	N/A	N/A	N/A	N/A
		35 M	40.2 M	IUD	LNG	10/14	N/A	N/A	N/A	N/A	N/A
Chen M 2016 [21]	16	32 (21–41) MD	N/A	Oral	MPA 250–500 mg	12/16	3/12	9/12	6/12	1/16	3/16
Pronin SM 2015 [22]	38	33 (28–42) MD	N/A	IUD	LNG	35/38	2/35	N/A	N/A	0/38	3/38
Shan BE 2013 [23]	12	25 M	29.25 (18–38) M	Oral + HR	MA 160 mg	10/12	3/10	N/A	N/A	0/12	2/12
Yu M 2009 [24]	17	29.9 M	N/A	Oral	MPA 100–500 mg	14/17	3/14	4/10	4/10	3/17	N/A
Kim N.K. 2024 [25]	124	35.2 M	26.8 M	Oral	MA 40–400 mg, MPA 10–500 mg	61/74	17/61	N/A	N/A	N/A	N/A
		34.2 M	28.3 M	IUD	LNG	27/37	2/27	N/A	N/A	N/A	N/A
		34.5 M	26.9 M	Oral + IUD	MA 40–400 mg, MPA 10–500 mg, LNG	11/13	2/11	N/A	N/A	N/A	N/A
Shikeli S 2020 [26]	18	31 MD	33 m	Oral	MA 160 mg	11/11	5/11	N/A	N/A	N/A	N/A
		31 MD	33 m	Oral + IUD	MA 160 mg, LNG	6/7	6/6	N/A	N/A	N/A	N/A
Chen J 2022 [27]	40	32 (21–42) MD	33.5 m	Oral	MA 160 mg, MPA 500 mg	16/19	3/16	3/9	2/9	N/A	N/A
		32 (21–42) MD	33.5 m	IUD + GnRHa	LNG, GnRHa	20/21	2/20	4/14	2/14	N/A	N/A
Akhavan S 2021 [28]	50	32.4 M	30 M	Oral	MA 160–320 mg	31/50	2/31	8/25	N/A	6/50	13/50
Fang F 2021 [29]	47	30 M	N/A	IUD	LNG	11/20	N/A	5/11	2/11	3/20	6/20
		30	N/A	Oral + IUD	LNG, MPA 10 mg	23/27	N/A	12/16	5/16	2/27	2/27
Novikova OV 2021 [30]	228	34 (20–46) MD	25.5 m	Oral	MPA 500 mg	34/37	N/A	N/A	N/A	N/A	N/A
		34 (20–46) MD	25.5 m	IUD	LNG	166/169	N/A	N/A	N/A	N/A	N/A
		34 (20–46) MD	25.5 m	IUD + GnRHa	LNG, Goserelin 3.6 mg	19/20	N/A	N/A	N/A	N/A	N/A
Ohyagi-Hara C 2015 [31]	11	34.2 (22–43) M	24 (16–47.3) M	Oral	MPA 400–600 mg	9/11	0/9	5/9	5/9	N/A	N/A
Minig L 2011 [32]	20	34 (22–40) M	21 (17–41) M	IUD + GnRHa	LNG, GnRHa	19/20	4/19	8/19	6/19	N/A	N/A
Kaku T 2001 [33]	18	29.3 (21–42) M	N/A	Oral	MPA 100–800 mg	13/18	2/13	5/11	4/11	2/18	3/18
Mitsuhashi A 2019 [34]	21	35 (26–44) MD	31 (15.4–50.8) m	Oral + Met	MPA 400–600 mg, Met 750–2250 mg	21/21	1/21	N/A	N/A	N/A	N/A
Ouasti S 2022 [35]	64	35 (19–47) MD	28 (6–123) m	Oral	Chlormadinone acetate	56/64	N/A	N/A	N/A	6/64	2/64
Casadio P 2020 [36]	46	32.2 M	27 M	Oral + HR	MA 160 mg	46/46	4/46	31/45	21/45	N/A	N/A
Giampaolino P 2019 [37]	55	25.9 M	35.1 M	IUD + HR	LNG	51/55	2/51	10/25	10/25	2/55	2/55
Goh CSY 2024 [15]	36	32 MD	32.8 (25.1–50.8) m	Oral	MA 160 mg	16/18	N/A	6/11	3/11	N/A	2/18
		32 (23–40) MD	35.5 (20.0–55.9) m	IUD	LNG	16/18	N/A	2/8	1/8	0/18	2/18
Tamauchi S 2024 [38]	56	34.2(19.4–44.6) MD	24.5(18–45) m	Oral	MPA 600 mg	53/56	27/56	26/36	17/36	N/A	3/56
Lee SH 2023 [39]	42	42.32 M	30.44 M	Oral	Norethisterone 20–40 mg Dydrogesterone 20 mg Megestrol acetate 160–320 mg	23/37	8/23	N/A	N/A	N/A	N/A
		34.2 M	36.4 M	IUD	LNG	5/5	0/5	N/A	N/A	0/5	N/A
Leone Roberti Maggiore U 2019 [40]	28	35.1 M	25 M	IUD	LNG	25/28	9/25	6/11	5/11	2/28	1/28
Yang B 2018 [41]	151	33.0 (21–54) MD	24.23 (17.07–37.95) m	Oral	MA 160 mg	78/82	N/A	N/A	N/A	N/A	N/A
		33.0 (21–54) MD	24.23 (17.07–37.95) m	Oral + Met	MA 160 mg, Met 1500 mg	63/69	N/A	N/A	N/A	N/A	N/A
Baek JS 2016 [42]	18	33 MD	23.1 m	Oral	MA 80–160 mg	16/18	2/16	N/A	N/A	N/A	N/A
Hubbs J 2013 [43]	114	45 (25–83) MD	36.1 (16.9–92.9) m	Oral	N/A	64/79	9/64	1/14	1/14	N/A	N/A
		47 (29–92) MD	50.8 (25–70) m	IUD	LNG	31/35	8/31	1/6	1/6	N/A	N/A
Kataoka H 2014 [44]	3	34 M	23 M	Oral	MPA 400–600 mg	3/3	0/3	1/3	1/3	N/A	N/A
Kong W 2022 [45]	219	33 M	26.33 M	Oral	MA 160 mg, MPA 250–500 mg	116/138	N/A	14/68	9/68	N/A	N/A
		32 M	27 M	Oral + Met	MA 160 mg, MPA 250–500 mg, Met 500 mg	76/81	N/A	10/47	6/47	N/A	N/A
Brownfoot F 2014 [46]	42	37 M	35 M	Oral	MPA	24/32	N/A	N/A	N/A	N/A	N/A
		37 M	35 M	IUD	LNG	8/10	N/A	N/A	N/A	N/A	N/A
Dagher C 2023 [47]	6	36.9 M	32.4	Oral	MA 160–320 mg	1/3	N/A	1/3	1/3	N/A	N/A
		37.7 M	40.7 M	Oral + IUD	MA 160–320 mg, LNG	2/3	N/A	0/3	0/3	N/A	N/A
Han A 2009 [48]	3	32.7 M	N/A	Oral	MA 80–160 mg, MPA 20–1000 mg	3/3	N/A	2/3	2/3	N/A	N/A
Koskas M 2012 [49]	14	34.5 M	N/A	Oral	N/A, MPA, MA, CA	12/14	N/A	6/12	5/12	N/A	N/A
Mentrikoski J 2012 [50]	6	28 (25–29) MD	N/A	Oral	N/A	3/6	N/A	N/A	N/A	1/6	2/6
Pal N 2018 [51]	15	45.6 (18.5–85.2) MD	45 (20–74) m	IUD	LNG	11/15	N/A	N/A	N/A	1/15	3/15
Raffone A 2021 [52]	37	36.1 (5.9) M	27.9 (7.6) M	IUD + HR	LNG	37/37	8/37	10/37	N/A	N/A	N/A
Randall T 1997 [53]	17	34.3 (25–39) MD	N/A	Oral	MA 40–160 mg, MPA 10 mg	16/17	2/16	2/16	2/16	N/A	1/17
Ricciardi E 2012 [54]	14	32 M	N/A	Oral	MA 80–160 mg, MPA 500–1000 mg	11/14	N/A	4/11	N/A	N/A	N/A
Ushijima K 2023 [7]	159	35 (19–44) MD	24.5 (15.2–50.8) m	Oral	MPA 400–600 mg	112/137	N/A	N/A	N/A	N/A	N/A
		35 (19–44) MD	24.5 (15.2–50.8) m	Oral + Met	MPA 400–600 mg, Met 750–2500 mg	21/22	N/A	N/A	N/A	N/A	N/A
Wheeler D 2007 [55]	11	35.3 M	N/A	Oral	N/A	4/9	N/A	N/A	N/A	1/9	4/9
		35.3 M	N/A	IUD	LNG	2/2	N/A	N/A	N/A	0/2	0/2
Yamagami W 2018 [56]	65	36 (20–45) MD	21.3 (16.4–40.2) m	Oral	MPA 400–600 mg	64/65	27/64	19/65	N/A	N/A	N/A
Zhang X 2023 [57]	20	33.4 ± 1.1 M	24.0 ± 1.2 M	Oral	MPA 500 mg	16/20	1/16	N/A	N/A	2/20	2/20
Minaguchi T 2007 [58]	12	37.7 M	N/A	Oral	MPA 400–600 mg	11/12	4/11	5/9	2/9	N/A	N/A
Shan W 2014 [59]	16	34 ± 7.1 M	N/A	Oral	MA 160 mg	2/8	N/A	N/A	N/A	2/8	4/8
		36.4 ± 4.2 M	N/A	Oral + Met	MA 160 mg, Met 1500 mg	6/8	N/A	N/A	N/A	0/8	2/8
Signorelli M 2009 [60]	10	N/A	N/A	Oral	Natural progesterone 200 mg	1/10	N/A	N/A	N/A	N/A	4/10
Kim MK 2016 [61]	15	42.67 M	N/A	IUD	LNG	15/15	N/A	N/A	N/A	N/A	N/A

^1^ Age is measured in years, M = mean, m = median, ^2^ BMI is measured as (kg/m^2^), M = mean, MD = median, ^3^ Therapeutic methods are: Oral = oral progestins, IUD = intrauterine device, GnRHa = gonadotropin-releasing hormone agonists, HR = Hysteroscopic resection, Met = metformin ^4^ Therapeutic regimens are MA = Megestrol acetate, MPA = Medroxyprogesterone acetate, LNG = Levonorgestrel, N/A = Norethisterone acetate, CA = Clomiphene acetate, ^5^ CR = complete response, ^6^ Recurrence rate, ^7^ Pregnancy rate, ^8^ Birth rate are calculated from the patients reached CR, ^9^ Partial response, ^10^ No response.

## Data Availability

This study is based exclusively on publicly available data; no new data were generated or analyzed in support of this research. Upon reasonable request, we are happy to provide any further information regarding the preparation of this manuscript.

## Supplementary Materials

The following supporting information can be downloaded at https://www.mdpi.com/article/10.3390/cancers17243966/s1, Figure S1: Forest plots of partial response and no-response rate; Figure S2: Funnel plots; Figure S3: Forest plots of recurrence rates stratified by follow-up duration; Table S1: GRADE Summary of Findings; Table S2: Risk of bias assessment of included studies; Table S3: List of excluded studies with reasons for exclusion; Table S4: PRISMA 2000 Checklist; Table S5: PRISMA 2000 for Abstracts Checklist.

## Abbreviations

The following abbreviations are used in this manuscript:

AEH	Atypical endometrial hyperplasia
CR	Complete response
EC	Endometrial carcinoma
EIN	Endometrial intraepithelial neoplasia
FST	Fertility-sparing statement
GnRHa	Gonadotropin-releasing hormone analogue
HR	Hysteroscopic resection
LBR	Live birth rate
LNG-IUD	Levonorgestrel-releasing intrauterine device
MA	Megestrol acetate
MPA	Medroxyprogesterone acetate
NR	No response
OP	Oral progestin
PR	Partial response
RCT	Randomized controlled trial
RR	Recurrence rate
