# Supplementary material for: Oncologic and Reproductive Outcomes After Fertility-Sparing Treatments for Endometrial Hyperplasia with Atypia: A Systematic Review and Meta-Analysis
Source: Cancers (Basel). 2025 Dec 12;17(24):3966. doi: 10.3390/cancers17243966 (PMC12730597; doi:10.3390/cancers17243966)
Supplement: Supplementary file 1 [file cancers-17-03966-s001.zip › cancers-4026061-supplementary.pdf]

cancers

MDPI

Supplementary Materials: Oncologic and Reproductive Outcomes After Fertility-Sparing Treatments for Endometrial Hyperplasia with Atypia: A Systematic Review and Meta-Analysis

Pál Sebok, Márton Keszthelyi, Balázs Vida, Lotti Lőczi, Barbara Sebők, Petra Merkely, Nándor Ács, Attila Keszthelyi, Szabolcs Várbíró, Balázs Lintner and Richárd Tóth

This table presents the certainty of evidence assessments for primary and secondary outcomes, including complete response, recurrence, pregnancy, live birth, partial response, and no response. For each intervention comparison, the table reports the number of studies, study design, risk of bias, inconsistency, indirectness, imprecision, and other considerations, alongside the total number of patients, relative effect estimates (with 95% confidence intervals), absolute risks, and final certainty rating. Critical and important outcomes are labeled according to their clinical relevance.

Table S1. GRADE Summary of Findings tables for fertility-sparing treatments in atypical endometrial hyperplasia (AEH).

	Certainty assessment							Effect				
Intervention	№ of studies	Study design	Risk of bias	Inconsistency	Indirectness	Imprecision	Other considerations	No. of patients	Relative (95% CI)	Absolute (95% CI)	Certainty	Importance
**Complete Response**												
LNG-IUD vs LNG-IUD + GnRHa	15	Randomised and nonrandomised studies	Not serious	Not serious	Not serious	Not serious	Downgraded due to marked imbalance in participant numbers between intervention arms, which may bias the pooled effect estimates.	467	0.95 (0.88–1.02)	88% vs 95%	Moderate	Critical
Oral Progestin vs. LNG-IUD	41	Randomised and nonrandomised studies	Not serious	Not serious	Not serious	Not serious	No additional considerations; no downgrading applied.	1748	0.88 (0.85–0.92)	80% vs 88%	High	Critical
LNG-IUD vs Oral Progestin + Hysteroscopy	16	Randomised and nonrandomised studies	Not serious	Not serious	Not serious	Not serious	Downgraded due to marked imbalance in participant numbers between intervention arms, which may bias the pooled effect estimates.	491	0.94 (0.88–0.99)	88% vs 96%	Moderate	Critical

	Certainty assessment							Effect				
Intervention	№ of studies	Study design	Risk of bias	Inconsistency	Indirectness	Imprecision	Other considerations	No. of patients	Relative (95% CI)	Absolute (95% CI)	Certainty	Importance
LNG-IUD vs Oral Progestin + IUD	15	Randomised and nonrandomised studies	Not serious	Not serious	Not serious	Not serious	Downgraded due to minimal differences between intervention effects, with effect estimates too close to distinguish a clinically relevant difference.	456	1.06 (0.94–1.20)	88% vs 86%	Moderate	Critical
LNG-IUD vs Oral Progestin + Metformin	20	Randomised and nonrandomised studies	Not serious	Not serious	Not serious	Not serious	Downgraded due to minimal differences between intervention effects, with effect estimates too close to distinguish a clinically relevant difference.	685	1.02 (0.96–1.08)	88% vs 89%	Moderate	Critical
Oral Progestin vs. LNG-IUD + GnRHa	38	Randomised and nonrandomised studies	Not serious	Not serious	Not serious	Not serious	Downgraded due to marked imbalance in participant numbers between intervention arms, which may bias the pooled effect estimates.	1403	0.84 (0.78–0.906)	80% vs 95%	Moderate	Critical
Oral Progestin + Hysteroscopy vs. LNG-IUD + GnRHa	6	Nonrandomised studies	Not serious	Not serious	Not serious	Not serious	Downgraded due to the limited number of participants, which reduces the precision and generalizability of the effect estimate.	146	1.02 (0.94–1.1)	96% vs 95%	Low	Critical
Oral Progestin + IUD vs. LNG-IUD + GnRHa	7	Randomised and nonrandomised studies	Not serious	Not serious	Not serious	Not serious	Downgraded due to the limited number of participants, which reduces the precision and generalizability of the effect estimate.	111	0.9 (0.78–1.03)	86% vs 96%	Low	Critical
Oral Progestin + Metformin vs. LNG-IUD + GnRHa	10	Randomised and nonrandomised studies	Not serious	Not serious	Not serious	Not serious	No additional considerations; no downgrading applied.	338	0.94 (0.86–1.01)	89% vs 96%	Low	Critical
Oral Progestin vs Oral Progestin + Hysteroscopy	40	Randomised and nonrandomised studies	Not serious	Not serious	Not serious	Not serious	Downgraded due to marked imbalance in participant numbers between intervention arms, which may bias the pooled effect estimates.	1427	0.83 (0.78–0.87)	80% vs 96%	Moderate	Critical

	Certainty assessment							Effect				
Intervention	№ of studies	Study design	Risk of bias	Inconsistency	Indirectness	Imprecision	Other considerations	No. of patients	Relative (95% CI)	Absolute (95% CI)	Certainty	Importance
Oral Progestin vs Oral Progestin + IUD	38	Randomised and nonrandomised studies	Not serious	Not serious	Not serious	Not serious	Downgraded due to marked imbalance in participant numbers between intervention arms, which may bias the pooled effect estimates.	1392	0.94 (0.83–1.06)	80% vs 86%	Low	Critical
Oral Progestin vs Oral Progestin + Metformin	38	Randomised and nonrandomised studies	Not serious	Not serious	Not serious	Not serious	No additional considerations; no downgrading applied.	1619	0.90 (0.85–0.95)	80% vs 89%	High	Critical
Oral Progestin + Hysteroscopy vs Oral Progestin + IUD	7	Nonrandomised studies	Not serious	Not serious	Not serious	Not serious	Downgraded due to the limited number of participants, which reduces the precision and generalizability of the effect estimate.	135	1.13 (0.99–1.3)	96% vs 86%	Low	Critical
Oral Progestin + Hysteroscopy vs Oral Progestin + Metformin	10	Randomised and nonrandomised studies	Not serious	Not serious	Not serious	Not serious	No additional considerations; no downgrading applied.	362	1.10 (1.02–1.16)	96% vs 89%	Moderate	Critical
Oral Progestin + IUD vs Oral Progestin + Metformin	11	Randomised and nonrandomised studies	Not serious	Not serious	Not serious	Not serious	Downgraded due to minimal differences between intervention effects, with effect estimates too close to distinguish a clinically relevant difference.	327	0.96 (0.85–1.09)	86% vs 89%	Low	Critical
**Live birth rate**												
LNG-IUD vs Oral Progestin	19	Randomised and nonrandomised studies	Not serious	Not serious	Not serious	Not serious	Downgraded due to unexplained heterogeneity across studies, with inconsistent effect estimates and overlapping confidence intervals.	308	0.94 (0.51–1.71)	24% vs 29%	Moderate	Critical
**Pregnancy rate**												
LNG-IUD vs Oral Progestin	22	Randomised and nonrandomised studies	Not seriuos	Not seriuos	Not seriuos	Not seriuos	No additional considerations; no downgrading applied.	411	1.006 (0.66–1.54)	39% vs 38%	High	Critical

	Certainty assessment							Effect			
Intervention	№ of studies	Study design	Risk of bias	Inconsistency	Indirectness	Imprecision	Other considerations	No. of patients	Relative (95% CI)	Absolute (95% CI)	Certainty	Importance
**Recurrence rate**												
Oral progestin vs. LNG-IUD	21	Randomised and nonrandomised studies	Not serious	Not serious	Not serious	Not serious	No additional considerations; no downgrading applied.	603	1.6 (1.05-2.56)	22% vs 14%	High	Critical
LNG-IUD vs Oral Progestin + Hysteroscopic resection	9	Randomised and nonrandomised studies	Not serious	Not serious	Not serious	Not serious	Downgraded due to the limited number of participants, which reduces the precision and generalizability of the effect estimate.	204	1.24 (0.58–3.76)	14% vs 11%	Moderate	Critical
Oral Progestin vs Oral Progestin + Hysteroscopic resection	23	Randomised and nonrandomised studies	Not serious	Not serious	Not serious	Not serious	Downgraded due to marked imbalance in participant numbers between intervention arms, which may bias the pooled effect estimates.	561	1.99 (1.05–3.76)	22% vs 11%	Moderate	Critical
**No response**												
Oral Progestin vs. LNG-IUD	13	Randomised and nonrandomised studies	Not serious	Not serious	Not serious	Not serious	Downgraded due to unexplained heterogeneity across studies, with inconsistent effect estimates and overlapping confidence intervals.	442	1.23 (0.72 – 2.1)	16% vs 13%	Moderate	Important
**Partial response**												
Oral Progestin vs. LNG-IUD	18	Randomised and nonrandomised studies	Not serious	Not serious	Not serious	Not serious	Downgraded due to unexplained heterogeneity across studies, with inconsistent effect estimates and overlapping confidence intervals.	288	1.16 (0.5-2.72)	11% vs. 9.5%	Low	Important

Table S2. Risk of bias assessment of included studies. (A) Risk of bias evaluation of observational studies using the ROBINS-I tool. Domains assessed included: D1, bias due to confounding; D2, bias due to selection of participants; D3, bias in classification of interventions; D4, bias due to deviations from intended interventions; D5, bias due to missing data; D6, bias in measurement of outcomes; and D7, bias in selection of the reported result. Overall risk of bias judgments are summarized for each study (green = low risk; yellow = moderate risk). (B) Risk of bias evaluation of randomized controlled trials using the Cochrane RoB-2 tool. Domains assessed included: D1, bias arising from the randomization process; D2, bias due to deviations from intended interventions; D3, bias due to missing outcome data; D4, bias in measurement of the outcome; and D5, bias in selection of the reported result. Overall risk of bias judgments are summarized for each trial (green = low risk).

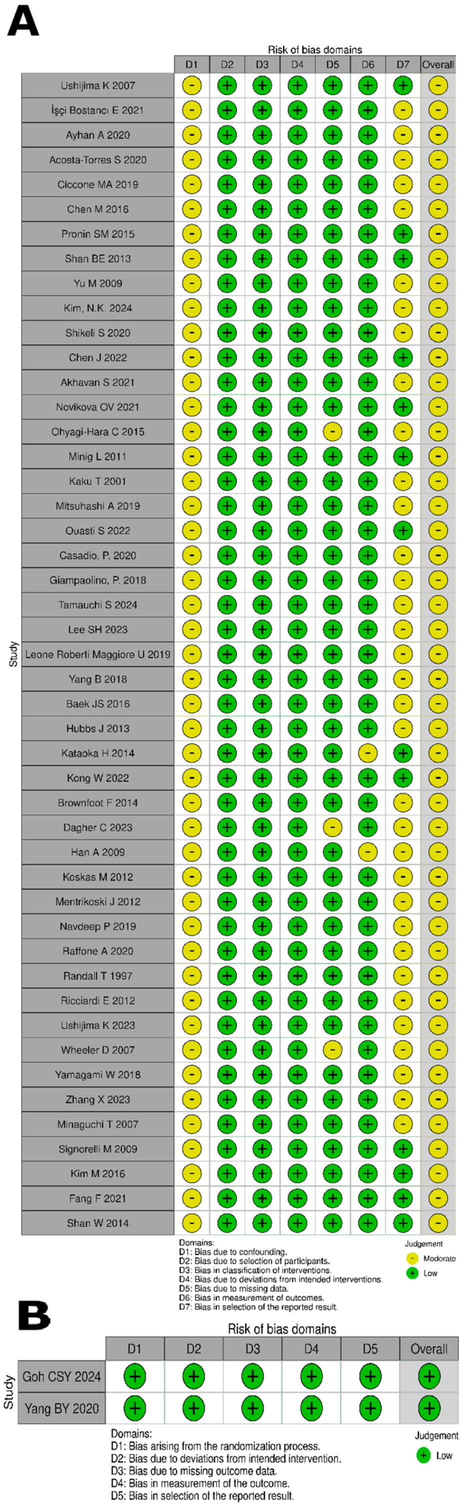

Table S3. List of excluded studies with reasons for exclusion. This table provides an overview of all articles that were assessed in full text but excluded from the systematic review, along with the specific reasons for exclusion (e.g., wrong intervention, wrong study design, no differentiation between interventions).

Author	Title	Reason for exclusion
**Yang 2019**	Treatment efficiency of comprehensive hysteroscopic evaluation and lesion resection combined with progestin therapy in young women with endometrial atypical hyperplasia and endometrial cancer.	Wrong intervention
**De Marzi 2015**	Hysteroscopic Resection in Fertility-Sparing Surgery for Atypical Hyperplasia and Endometrial Cancer: Safety and Efficacy.	Wrong population
**Andress 2021**	Fertility preserving management of early endometrial cancer in a patient cohort at the department of women's health at the university of Tuebingen	Wrong study design
**Chen 2022**	Oncological and reproductive outcomes for gonadotropin-releasing hormone agonist combined with aromatase inhibitors or levonorgestrel-releasing intra-uterine system in women with endometrial cancer or atypical endometrial hyperplasia	Wrong intervention
**Pashov 2012**	The combined GnRH-agonist and intrauterine levonorgestrel-releasing system treatment of complicated atypical hyperplasia and endometrial cancer: a pilot study	Wrong study design
**Tamauchi 2017**	Efficacy of medroxyprogesterone acetate treatment and retreatment for atypical endometrial hyperplasia and endometrial cancer	Duplication of results
**Atallah 2021**	The use of hysteroscopic endometrectomy in the conservative treatment of early endometrial cancer and atypical hyperplasia in fertile women	Wrong intervention
**Baker 2017**	Nonoperative management of atypical endometrial hyperplasia and grade 1 endometrial cancer with the levonorgestrel intrauterine device in medically ill post-menopausal women	Wrong population
**Fan 2021**	Analysis of pregnancy-associated factors after fertility-sparing therapy in young women with early stage endometrial cancer or atypical endometrial hyperplasia	Wrong study design
**Gonthier 2014**	Impact of obesity on the results of fertility-sparing management for atypical hyperplasia and grade 1 endometrial cancer	Wrong study design
**He 2020**	Oncologic and obstetrical outcomes after fertility-preserving retreatment in patients with recurrent atypical endometrial hyperplasia and endometrial cancer	Wrong population
**Donnez 2003**	Conservative treatment may be beneficial for young women with atypical endometrial hyperplasia or endometrial adenocarcinoma	Wrong intervention
**Janda 2021**	Complete pathological response following levonorgestrel intrauterine device in clinically stage 1 endometrial adenocarcinoma: Results of a randomized clinical trial	Wrong study design
**Kim 2017**	Fertility-Sparing Management Using Progestin for Young Women with Endometrial Cancer From a Population-Based Study	Wrong population

**Le Digabel 2006**	Hyperplasies atypiques et carcinomes de l'endomètre de stade I chez la femme jeune désirant une grossesse : le traitement conservateur est-il envisageable ? Résultats d'une étude multicentrique françaiseYoung women with atypical endometrial hyperplasia or endometrial adenocarcinoma stage I: will conservative treatment allow pregnancy? Results of a French multicentric survey	Wrong language
**Marnach 2017**	Oral Progestogens Versus Levonorgestrel-Releasing Intrauterine System for Treatment of Endometrial Intraepithelial Neoplasia	Wrong population
**Masciullo 2021**	Prognostic impact of hysteroscopic resection of endometrial atypical hyperplasia-endometrioid intraepithelial neoplasia and early-stage cancer in combination with megestrol acetate	Wrong population
**Sengal 2021**	Fibroblast Growth Factor Receptor 2 Isoforms Detected via Novel RNA ISH as Predictive Biomarkers for Progestin Therapy in Atypical Hyperplasia and Low-Grade Endometrial Cancer	Wrong study design
**Tock 2018**	Fertility Sparing Treatment in Patients With Early Stage Endometrial Cancer, Using a Combination of Surgery and GnRH Agonist: A Monocentric Retrospective Study and Review of the Literature	Wrong intervention
**van Gent MDJM 2016**	Exploring Morphologic and Molecular Aspects of Endometrial Cancer Under Progesterone Treatment in the Context of Fertility Preservation	Wrong study design
**Wang 2022**	[Significance of molecular classification in fertility-sparing treatment of endometrial carcinoma and atypical endometrial hyperplasia]	Wrong language
**Xi 2022**	Efficacy and pregnancy outcomes of hysteroscopic surgery combined with progestin as fertility-sparing therapy in patients with early stage endometrial cancer and atypical hyperplasia	Wrong study design
**Yang 2015**	Prognostic factors of regression and relapse of complex atypical hyperplasia and well-differentiated endometrioid carcinoma with conservative treatment	Wrong study design
**Varma 2007**	The effectiveness of a levonorgestrel-releasing intrauterine system (LNG-IUS) in the treatment of endometrial hyperplasia—A long-term follow-up study	Wrong population
**òrbo 2016**	HE4 is a novel tissue marker for therapy response and progestin resistance in medium- and low-risk endometrial hyperplasia	Wrong study design
**Von Minckwitz 2002**	Adjuvant endocrine treatment with medroxyprogesterone acetate or tamoxifen in stage I and II endometrial cancer—a multicentre, open, controlled, prospectively randomised trial	Wrong population
**Yuan 2022**	Metformin in Combination with Progesterone Improves the Pregnancy Rate for Patients with Early Endometrial Cancer	Wrong population
**Sletten 2019**	Significance of progesterone receptors (PR-A and PR-B) expression as predictors for relapse after successful therapy of endometrial hyperplasia: a retrospective cohort study	Wrong study design

**Wu 2022**	Clinical implications of morular metaplasia in fertility-preserving treatment for atypical endometrial hyperplasia and early endometrial carcinoma patients	Wrong population
**Yoshimura 2022**	Clinical Usefulness of Endometrial Cytology in Determining the Therapeutic Effect of Fertility Preserving Therapy	Wrong study design
**Liu 2022**	Efficacy of Levonorgestrel-intrauterine Releasing System Combined with Goserelin in Treatment of Atypical Endometrial Hyperplasia	Full text not available
**He 2021**	Maintenance Therapy Can Improve the Oncologic Prognosis and Obstetrical Outcome of Patients With Atypical Endometrial Hyperplasia and Endometrial Cancer After Fertility-Preserving Treatment: A Multicenter Retrospective Study	Wrong intervention
**Wang 2021**	Significance of serum and pathological biomarkers in fertility-sparing treatment for endometrial cancer or atypical hyperplasia: a retrospective cohort study	Wrong intervention
**Li 2021**	Insulin Resistance and Metabolic Syndrome Increase the Risk of Relapse For Fertility Preserving Treatment in Atypical Endometrial Hyperplasia and Early Endometrial Cancer Patients	Wrong study design
**Shan 2021**	Effect and Management of Excess Weight in the Context of Fertility-Sparing Treatments in Patients With Atypical Endometrial Hyperplasia and Endometrial Cancer: 8-Year Experience of 227 Cases	Wrong outcomes
**Wang 2021**	Fertility-preserving treatment outcome in endometrial cancer or atypical hyperplasia patients with polycystic ovary syndrome	Wrong population
**Piatek 2021**	The results of different fertility-sparing treatment modalities and obstetric outcomes in patients with early endometrial cancer and atypical endometrial hyperplasia: Case series of 30 patients and systematic review	Wrong population
**Sengal 2021**	Fibroblast growth factor receptor 2 isoforms detected via novel rna ish as predictive biomarkers for progestin therapy in atypical hyperplasia and low-grade endometrial cancer	Wrong outcomes
**Westin 2021**	Prospective phase II trial of levonorgestrel intrauterine device: nonsurgical approach for complex atypical hyperplasia and early-stage endometrial cancer	Wrong population
**Matsuo 2020**	Route-specific association of progestin therapy and concurrent metformin use in obese women with complex atypical hyperplasia	Wrong population
**Kim 2020**	Comparison of diagnostic accuracy between endometrial curettage and aspiration biopsy in patients treated with progestin for endometrial hyperplasia: A Korean gynecologic oncology group study	Wrong outcomes
**Mandelbaum 2020**	Progestin therapy for obese women with complex atypical hyperplasia: levonorgestrel-releasing intrauterine device vs systemic therapy	Wrong population
**Behrouzi 2020**	Baseline serum HE4 but not tissue HE4 expression predicts response to the levonorgestrel-releasing intrauterine system in atypical hyperplasia and early stage endometrial cancer	Wrong population

**Wang 2019**	Impact of treatment duration in fertility-preserving management of endometrial cancer or atypical endometrial hyperplasia	Wrong study design
**Yang 2019**	Treatment efficiency of comprehensive hysteroscopic evaluation and lesion resection combined with progestin therapy in young women with endometrial atypical hyperplasia and endometrial cancer	Wrong study design
**Kim 2018**	Fertility-Sparing Management Using Progestin for Young Women with Endometrial Cancer From a Population-Based Study	Wrong population
**Zhou 2017**	Gonadotropin-releasing hormone agonist combined with a levonorgestrel-releasing intrauterine system or letrozole for fertility-preserving treatment of endometrial carcinoma and complex atypical hyperplasia in young women	Wrong study design
**Laurelli 2016**	Long-Term Oncologic and Reproductive Outcomes in Young Women with Early Endometrial Cancer Conservatively Treated: A Prospective Study and Literature Update	Wrong population
**Yang 2015**	Prognostic factors of regression and relapse of complex atypical hyperplasia and well-differentiated endometrioid carcinoma with conservative treatment	Wrong intervention
**Zhang 2015**	Dual-specificity phosphatase 6 predicts the sensitivity of progestin therapy for atypical endometrial hyperplasia	Wrong outcomes
**Gonthier 2014**	Impact of obesity on the results of fertility-sparing management for atypical hyperplasia and grade 1 endometrial cancer	Wrong intervention
**Gunderson 2014**	Pathologic features associated with resolution of complex atypical hyperplasia and grade 1 endometrial adenocarcinoma after progestin therapy	Wrong study design
**Simpson 2014**	Fertility sparing treatment of complex atypical hyperplasia and low grade endometrial cancer using oral progestin	Wrong population
**Cade 2013**	Long-term outcomes after progestogen treatment for early endometrial cancer	Wrong population
**Goncharenko 2013**	Predictive diagnosis of endometrial hyperplasia and personalized therapeutic strategy in women of fertile age	Wrong outcomes
**Kim 2013**	Comparison of dilatation & curettage and endometrial aspiration biopsy accuracy in patients treated with high-dose oral progestin plus levonorgestrel intrauterine system for early-stage endometrial cancer	Wrong population
**Gallos 2013**	Predictive ability of estrogen receptor (ER), progesterone receptor (PR), COX-2, Mlh1, and Bcl-2 expressions for regression and relapse of endometrial hyperplasia treated with LNG-IUS: A prospective cohort study	Wrong outcomes
**Gallos 2013**	LNG-IUS vs oral progestogen treatment for endometrial hyperplasia: A long-term comparative cohort study	Wrong population
**Bakkum-Gamez 2012**	Conservative management of atypical hyperplasia and grade i endometrial carcinoma: Review of the literature and presentation of a series	Wrong intervention
**Upson 2012**	Biomarkers of progestin therapy resistance and endometrial hyperplasia progression	Wrong outcomes

**Dursun 2012**	A Turkish Gynecologic Oncology Group study of fertility-sparing treatment for early-stage endometrial cancer	Wrong population
**Perri 2011**	Prolonged conservative treatment of endometrial cancer patients: More than 1 pregnancy can be achieved	Wrong population
**Haoula 2011**	Levonorgestrel intra-uterine system as a treatment option for complex endometrial hyperplasia	Wrong population
**òrbo 2010**	Down-regulated progesterone receptor A and B coinciding with successful treatment of endometrial hyperplasia by the levonorgestrel impregnated intrauterine system	Wrong outcomes
**Cade 2010**	Progestogen treatment options for early endometrial cancer	Wrong population
**Vereide 2005**	Bcl-2, BAX, and apoptosis in endometrial hyperplasia after high dose gestagen therapy: A comparison of responses in patients treated with intrauterine levonorgestrel and systemic medroxyprogesterone	Wrong population
**Montz 2002**	Intrauterine progesterone treatment of early endometrial cancer	Wrong population
**Lago 2022**	Fertility sparing treatment in patients with endometrial cancer (FERT-ENC): a multicentric retrospective study from the Spanish Investigational Network Gynecologic Oncology Group (SPAIN-GOG)	Wrong population
**Chung 2021**	Mismatch repair status influences response to fertility-sparing treatment of endometrial cancer	Wrong population
**Kudesia 2013**	Reproductive and oncologic outcomes after progestin therapy forendometrial complex atypical hyperplasia or carcinoma	Wrong study design
**Greenwald 2016**	Does hormonal therapy for fertility preservation affect the survival ofyoung women with early-stage endometrial cancer?	Wrong population
**Yin 2022**	Clinical outcomes of levonorgestrel-releasing intrauterine device present during controlled ovarian stimulation in patients with early stage endometrioid adenocarcinoma and atypical endometrial hyperplasia after fertility-sparing treatments: 10-year experience in 1 tertiary hospital in China	Wrong study design
**Vereide 2006**	Effect of levonorgestrel IUD and oral medroxyprogesterone acetate on glandular and stromal progesterone receptors (PRA and PRB), and estrogen receptors (ER-alpha and ER-beta) in human endometrial hyperplasia	Wrong population
**Marnach 2017**	Oral Progestogens vs Levonorgestrel-Releasing Intrauterine System for Treatment of Endometrial Intraepithelial Neoplasia<sup/>	Wrong population
**Wu 2023**	Impacts of ovarian reserve on conservative treatment for endometrial cancer and atypical hyperplasia	Wrong study design
**Oishi 2023**	Obstetric outcomes after medroxyprogesterone acetate treatment for early stage endometrial cancer or atypical endometrial hyperplasia: a single hospital-based study	Wrong outcomes
**Barr 2023**	Serum HE4 predicts progestin treatment response in endometrial cancer and atypical hyperplasia: A prognostic study	Wrong outcomes
**Chaudhari 2023**	Comparison of Mirena and Liletta levonorgestrel intrauterine devices for the treatment of endometrial	Wrong study design

	intraepithelial neoplasia and grade 1 endometrioid endometrial cancer	
**Xue 2023**	PTEN mutation predicts unfavorable fertility preserving treatment outcome in the young patients with endometrioid endometrial cancer and atypical hyperplasia	Wrong outcomes
**Xi 2023**	Efficacy and pregnancy outcomes of hysteroscopic surgery combined with progestin as fertility-sparing therapy in patients with early stage endometrial cancer and atypical hyperplasia	Wrong study design
**Wang 2023**	Characteristics of molecular classification in 52 endometrial cancer and atypical hyperplasia patients receiving fertility-sparing treatment	Wrong population
**Ga 2023**	Prognosis of patients with endometrial cancer or atypical endometrial hyperplasia after complete remission with fertility-sparing therapy	Wrong outcomes
**Fu 2023**	Postoperative Adjuvant Treatment in Women with Stage i Endometrial Cancer: A Retrospective Study	Wrong population
**Kudesia 2014**	Reproductive and oncologic outcomes after progestin therapy for endometrial complex atypical hyperplasia or carcinoma	Wrong study design
**Chung 2019**	Oncologic and pregnancy outcomes with fertility-sparing management for early endometrial cancer in young women	Wrong population
**Milishkevich 2022**	The results of fertility-sparing treatment and obstetric outcomes in patients with atypical endometrial hyperplasia and early endometrial cancer: a case series from belarus	Wrong study design
**Lv 2023**	Efficacy of fertility-sparing treatment with LNG-IUS is associated with different ProMisE subtypes of endometrial carcinoma or atypical endometrial hyperplasia	Wrong outcomes
**Lin 2024**	DNA methylation profiling identifies subset of lowgrade endometrial neoplasms with poor response to progestin therapy	Wrong population
**Perri 2011**	Prolonged Conservative Treatment of Endometrial Cancer Patients More Than 1 Pregnancy Can Be Achieved	Wrong study design
**Kim 2013**	Combined medroxyprogesterone acetate/levonorgestrel-intrauterine system treatment in young women with early-stage endometrial cancer	Wrong population
**Kim 2019**	Six months response rate of combined oral medroxyprogesterone/levonorgestrel-intrauterine system for early-stage endometrial cancer in young women: a Korean Gynecologic-Oncology Group Study	Wrong population

Table S4. PRISMA 2000 Checklist.

Section and Topic	Item #	Checklist item	Location where item is reported
**TITLE**			
Title	1	Identify the report as a systematic review.	P1
**ABSTRACT**			
Abstract	2	See the PRISMA 2020 for Abstracts checklist.	P2, Supplemental Table 5.
**INTRODUCTION**			
Rationale	3	Describe the rationale for the review in the context of existing knowledge.	P4
Objectives	4	Provide an explicit statement of the objective(s) or question(s) the review addresses.	P4-5
**METHODS**			
Eligibility criteria	5	Specify the inclusion and exclusion criteria for the review and how studies were grouped for the syntheses.	P5
Information sources	6	Specify all databases, registers, websites, organisations, reference lists and other sources searched or consulted to identify studies. Specify the date when each source was last searched or consulted.	P5
Search strategy	7	Present the full search strategies for all databases, registers and websites, including any filters and limits used.	P5
Selection process	8	Specify the methods used to decide whether a study met the inclusion criteria of the review, including how many reviewers screened each record and each report retrieved, whether they worked independently, and if applicable, details of automation tools used in the process.	P5
Data collection process	9	Specify the methods used to collect data from reports, including how many reviewers collected data from each report, whether they worked independently, any processes for obtaining or confirming data from study investigators, and if applicable, details of automation tools used in the process.	P6
Data items	10a	List and define all outcomes for which data were sought. Specify whether all results that were compatible with each outcome domain in each study were sought (e.g. for all measures, time points, analyses), and if not, the methods used to decide which results to collect.	P6
	10b	List and define all other variables for which data were sought (e.g. participant and intervention characteristics, funding sources). Describe any assumptions made about any missing or unclear information.	P6
Study risk of bias assessment	11	Specify the methods used to assess risk of bias in the included studies, including details of the tool(s) used, how many reviewers assessed each study and whether they worked independently, and if applicable, details of automation tools used in the process.	P6-7
Effect measures	12	Specify for each outcome the effect measure(s) (e.g. risk ratio, mean difference) used in the synthesis or presentation of results.	P6-7
Synthesis methods	13a	Describe the processes used to decide which studies were eligible for each synthesis (e.g. tabulating the study intervention characteristics and comparing against the planned groups for each synthesis (item #5)).	P6-7
	13b	Describe any methods required to prepare the data for presentation or synthesis, such as handling of missing summary statistics, or data conversions.	P6-8
	13c	Describe any methods used to tabulate or visually display results of individual studies and syntheses.	P7-8
	13d	Describe any methods used to synthesize results and provide a rationale for the choice(s). If meta-analysis was performed, describe the model(s), method(s) to identify the presence and extent of statistical heterogeneity, and software package(s) used.	P7-8
	13e	Describe any methods used to explore possible causes of heterogeneity among study results (e.g. subgroup analysis, meta-regression).	P7-8, 19
	13f	Describe any sensitivity analyses conducted to assess robustness of the synthesized results.	P20

Section and Topic	Item #	Checklist item	Location where item is reported
Reporting bias assessment	14	Describe any methods used to assess risk of bias due to missing results in a synthesis (arising from reporting biases).	P7, 19
Certainty assessment	15	Describe any methods used to assess certainty (or confidence) in the body of evidence for an outcome.	P7, 19
**RESULTS**			
Study selection	16a	Describe the results of the search and selection process, from the number of records identified in the search to the number of studies included in the review, ideally using a flow diagram.	P6, 8
	16b	Cite studies that might appear to meet the inclusion criteria, but which were excluded, and explain why they were excluded.	Supplemental Table 3.
Study characteristics	17	Cite each included study and present its characteristics.	Table 1, P13
Risk of bias in studies	18	Present assessments of risk of bias for each included study.	Supplemental Table 2
Results of individual studies	19	For all outcomes, present, for each study: (a) summary statistics for each group (where appropriate) and (b) an effect estimate and its precision (e.g. confidence/credible interval), ideally using structured tables or plots.	Figure 2-5., Supplemental Figure 1
Results of syntheses	20a	For each synthesis, briefly summarise the characteristics and risk of bias among contributing studies.	P13-19
	20b	Present results of all statistical syntheses conducted. If meta-analysis was done, present for each the summary estimate and its precision (e.g. confidence/credible interval) and measures of statistical heterogeneity. If comparing groups, describe the direction of the effect.	P13-19
	20c	Present results of all investigations of possible causes of heterogeneity among study results.	P19
	20d	Present results of all sensitivity analyses conducted to assess the robustness of the synthesized results.	P20
Reporting biases	21	Present assessments of risk of bias due to missing results (arising from reporting biases) for each synthesis assessed.	P20, Supplemental Table 2
Certainty of evidence	22	Present assessments of certainty (or confidence) in the body of evidence for each outcome assessed.	P19, Supplemental Table 1
**DISCUSSION**			
Discussion	23a	Provide a general interpretation of the results in the context of other evidence.	P20-24
	23b	Discuss any limitations of the evidence included in the review.	P24
	23c	Discuss any limitations of the review processes used.	P24
	23d	Discuss implications of the results for practice, policy, and future research.	P23-24
**OTHER INFORMATION**			
Registration and protocol	24a	Provide registration information for the review, including register name and registration number, or state that the review was not registered.	P2
	24b	Indicate where the review protocol can be accessed, or state that a protocol was not prepared.	P2
	24c	Describe and explain any amendments to information provided at registration or in the protocol.	P7-8
Support	25	Describe sources of financial or non-financial support for the review, and the role of the funders or sponsors in the review.	P25
Competing interests	26	Declare any competing interests of review authors.	P25

Section and Topic	Item #	Checklist item	Location where item is reported
Availability of data, code and other materials	27	Report which of the following are publicly available and where they can be found: template data collection forms; data extracted from included studies; data used for all analyses; analytic code; any other materials used in the review.	P25

Table S5. PRISMA 2000 for Abstracts Checklist.

Section and Topic	Item #	Checklist item	Reported (Yes/No)
**TITLE**			
Title	1	Identify the report as a systematic review.	Yes
**BACKGROUND**			
Objectives	2	Provide an explicit statement of the main objective(s) or question(s) the review addresses.	Yes
**METHODS**			
Eligibility criteria	3	Specify the inclusion and exclusion criteria for the review.	Yes
Information sources	4	Specify the information sources (e.g. databases, registers) used to identify studies and the date when each was last searched.	Yes
Risk of bias	5	Specify the methods used to assess risk of bias in the included studies.	Yes
Synthesis of results	6	Specify the methods used to present and synthesise results.	Yes
**RESULTS**			
Included studies	7	Give the total number of included studies and participants and summarise relevant characteristics of studies.	Yes
Synthesis of results	8	Present results for main outcomes, preferably indicating the number of included studies and participants for each. If meta-analysis was done, report the summary estimate and confidence/credible interval. If comparing groups, indicate the direction of the effect (i.e. which group is favoured).	Yes
**DISCUSSION**			
Limitations of evidence	9	Provide a brief summary of the limitations of the evidence included in the review (e.g. study risk of bias, inconsistency and imprecision).	Yes
Interpretation	10	Provide a general interpretation of the results and important implications.	Yes
**OTHER**			
Funding	11	Specify the primary source of funding for the review.	Yes
Registration	12	Provide the register name and registration number.	Yes

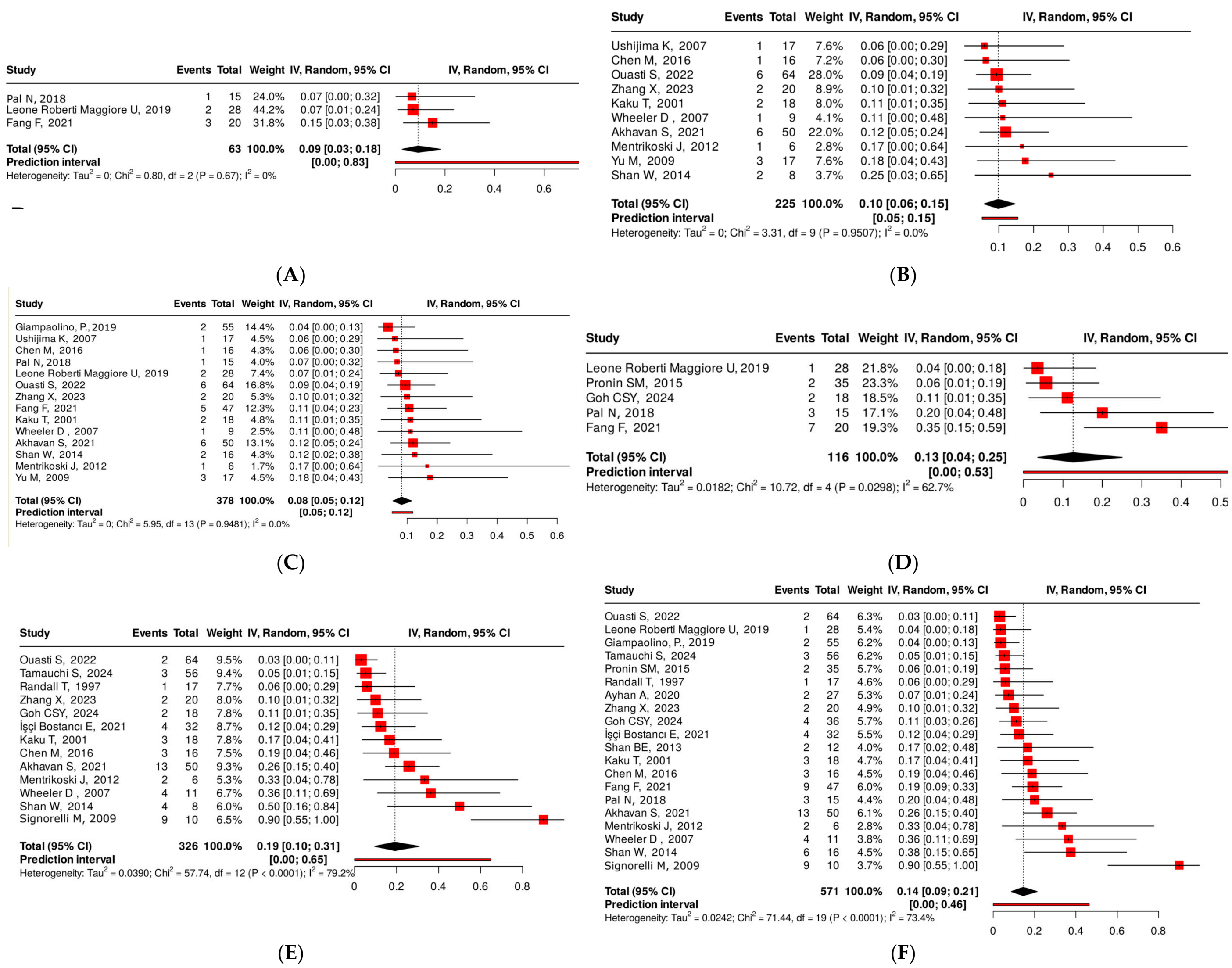

Figure S1. Forest plots of partial and no response rate.

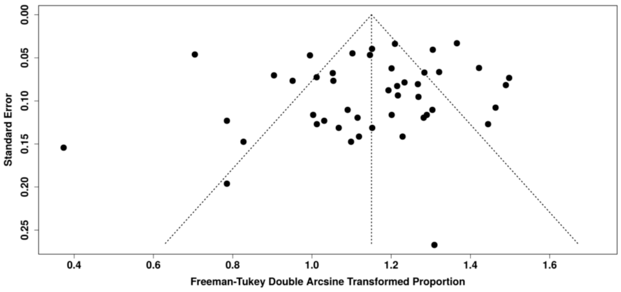

(**A**)

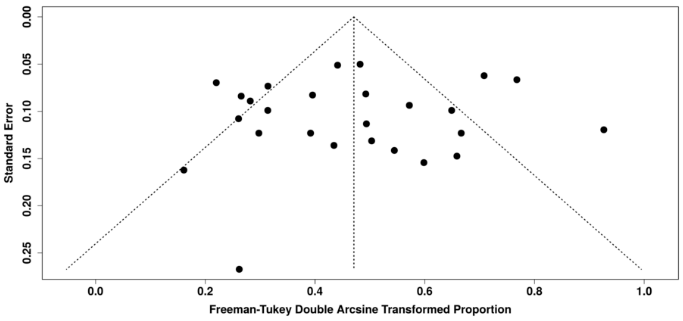

(**B**)

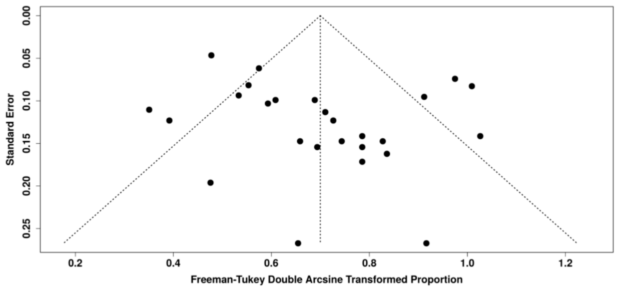

(**C**)

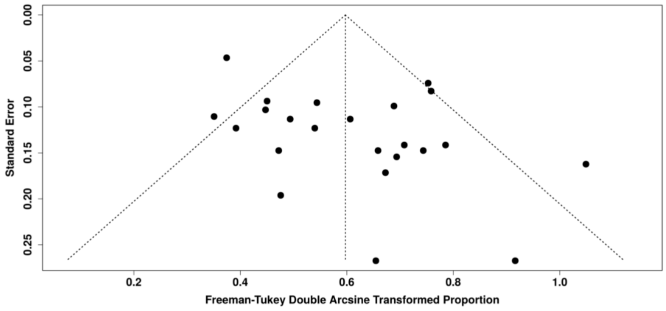

(**D**)

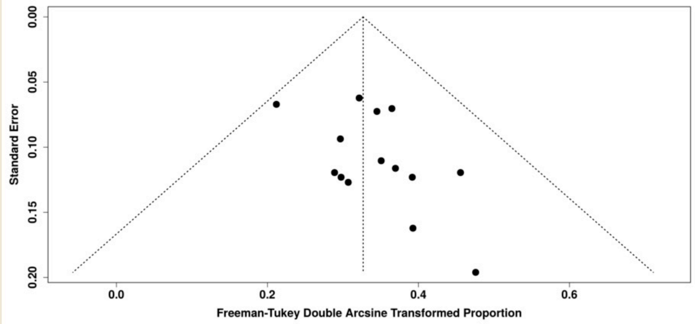

(**E**)

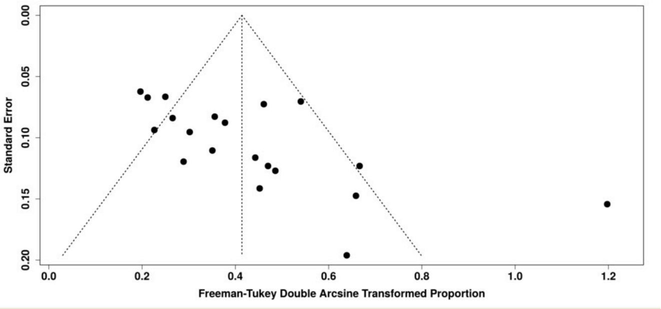

(**F**)

Figure S2. Funnel plots.

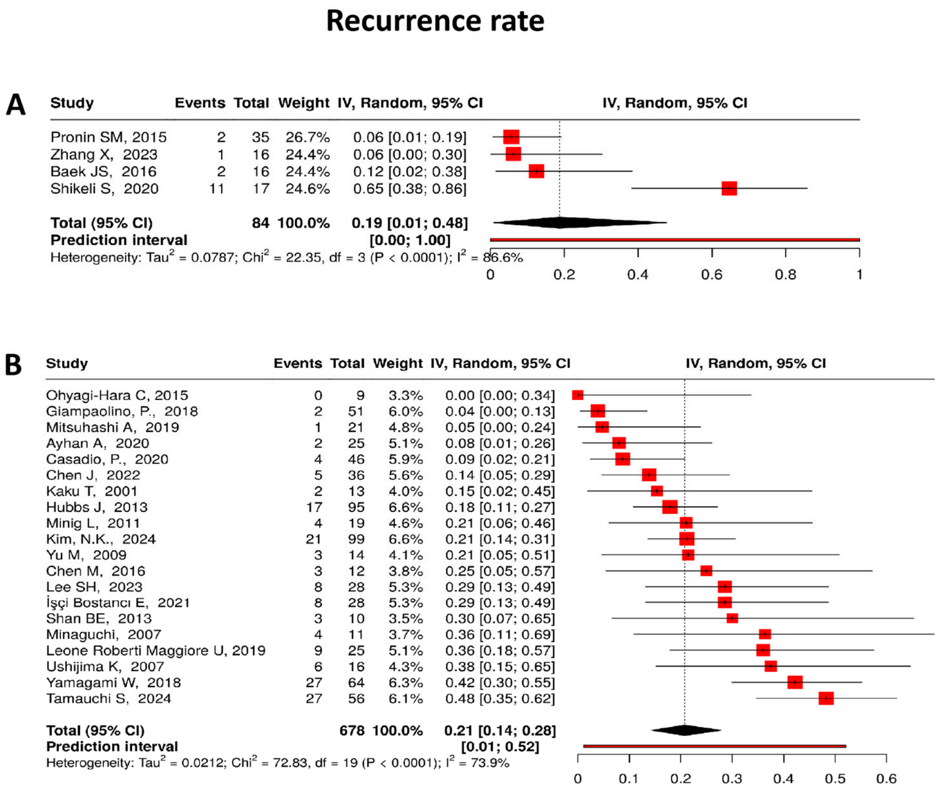

A: Recurrence rate in studies with less than 24 months of follow-up, **B:** Recurrence rate in studies with at least 24 months of follow-up

Figure S3. Forest plots of recurrence rates stratified by follow-up duration.
